# A comprehensive tool box for large animal studies of intervertebral disc degeneration

**DOI:** 10.1002/jsp2.1162

**Published:** 2021-06-14

**Authors:** Naomi N. Lee, Elias Salzer, Frances C. Bach, Andres F. Bonilla, James L. Cook, Zulma Gazit, Sibylle Grad, Keita Ito, Lachlan J. Smith, Andrea Vernengo, Hans‐Joachim Wilke, Julie B. Engiles, Marianna A. Tryfonidou

**Affiliations:** ^1^ Thompson Laboratory for Regenerative Orthopaedics University of Missouri Columbia Missouri USA; ^2^ Orthopaedic Biomechanics, Department of Biomedical Engineering Eindhoven University of Technology Eindhoven The Netherlands; ^3^ Department of Clinical Sciences, Faculty of Veterinary Medicine Utrecht University Utrecht The Netherlands; ^4^ Preclinical Surgical Research Laboratory, Department of Clinical Sciences Colorado State University Colorado USA; ^5^ Department of Surgery Cedars‐Sinai Medical Center Los Angeles California USA; ^6^ AO Research Institute Davos Davos Switzerland; ^7^ Departments of Neurosurgery and Orthopaedic Surgery University of Pennsylvania Philadelphia Pennsylvania USA; ^8^ Department of Chemical Engineering Rowan University Glassboro New Jersey USA; ^9^ Institute of Orthopaedic Research and Biomechanics University Hospital Ulm Ulm Germany; ^10^ Department of Pathobiology, New Bolton Center, School of Veterinary Medicine University of Pennsylvania Kennett Square Pennsylvania USA

**Keywords:** biomechanical testing, clinical assessment, disc disease, dog, goat, histopathology, intervertebral disc, low back pain, neck pain, pig, sheep, spine disorders, spine research

## Abstract

Preclinical studies involving large animal models aim to recapitulate the clinical situation as much as possible and bridge the gap from benchtop to bedside. To date, studies investigating intervertebral disc (IVD) degeneration and regeneration in large animal models have utilized a wide spectrum of methodologies for outcome evaluation. This paper aims to consolidate available knowledge, expertise, and experience in large animal preclinical models of IVD degeneration to create a comprehensive tool box of anatomical and functional outcomes. Herein, we present a Large Animal IVD Scoring Algorithm based on three scales: macroscopic (gross morphology, imaging, and biomechanics), microscopic (histological, biochemical, and biomolecular analyses), and clinical (neurologic state, mobility, and pain). The proposed algorithm encompasses a stepwise evaluation on all three scales, including spinal pain assessment, and relevant structural and functional components of IVD health and disease. This comprehensive tool box was designed for four commonly used preclinical large animal models (dog, pig, goat, and sheep) in order to facilitate standardization and applicability. Furthermore, it is intended to facilitate comparison across studies while discerning relevant differences between species within the context of outcomes with the goal to enhance veterinary clinical relevance as well. Current major challenges in pre‐clinical large animal models for IVD regeneration are highlighted and insights into future directions that may improve the understanding of the underlying pathologies are discussed. As such, the IVD research community can deepen its exploration of the molecular, cellular, structural, and biomechanical changes that occur with IVD degeneration and regeneration, paving the path for clinically relevant therapeutic strategies.

## Graphical abstract

The basic tool box entailing clinical, macroscopic, and microscopic scale outcomes was developed to guide preclinical large animal studies in the field of disc‐related spinal pain. In depth understanding of the intricate relationship between molecular, cellular, structural, and biomechanical changes that occur with IVD degeneration and regeneration will help pave the path for clinically relevant therapeutic strategies in the field.

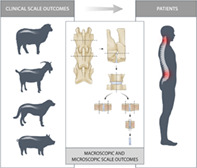

## 1 INTRODUCTION

Chronic low back pain has been reported as the leading cause of years lost to disability for the past three decades.^1^, ^2^ Intervertebral disc (IVD) degeneration is recognized in at least 40% of cases of symptomatic back pain.^3^, ^4^ Much effort has been aimed toward the development of more effective diagnostic, preventative, and therapeutic strategies for IVD degeneration and preclinical studies involving large animal models are still considered a critical translational tool. Advantages of large animal models include the use of whole‐organ/whole‐body systems, relative vertebra‐disc geometry,^5^biomechanics,^6^, ^7^ similar pathology, and clinically relevant outcome measures (reviewed by Thorpe et al^8^).

Most large animal models require an experimentally induced insult or injury to the IVD in order to create and study IVD degeneration, with each method for induction having specific advantages and disadvantages.^9^, ^10^, ^11^, ^12^ It is beyond the scope of this article to provide a detailed review of large animal IVD models, their strengths and weaknesses, and the extent to which these models reproduce all the characteristics of pathological human IVDs. Instead, the reader is referred to previous reviews on this topic.^9^, ^10^, ^11^, ^12^ Furthermore, there are species‐specific biological considerations, including overall size, age of skeletal maturation, spinal anatomy, and cellular composition in the nucleus pulposus (NP) (Table 1), and IVD biomechanics, and from a clinical perspective, differences in pain‐related behaviors. Within the particular species employed, confounding factors including sex (hormones), genetics, and susceptibility to developing spontaneous IVD conditions all need to be considered. Preclinical findings from experimental animal models can be supplemented with clinical studies involving client‐owned companion animals, for example, canine patients. Dogs develop spontaneous degenerative IVD diseases and manifest with well‐recognized clinical presentations. The diagnostics and treatment are closely comparable to those for humans (reviewed by Lee et al^13^). Although these studies are emerging, as yet their implementation is limited.

**TABLE 1:** Overview of experimental domesticated large animal models used in IVD studies

	Dog^b^	Pig	Sheep	Goat
Average expected lifespan	10–15 y	15‐20 y	10‐12 y	15‐18 y
Age at skeletal maturity^a^	NCD: 10‐11 mo^191^ CD: 8‐9 mo^191^	18 mo‐4 y	2‐3 y	2‐3 y
Bodyweight^c^	NCD: 20‐30 kg CD: less than 15 kg	Miniature: 30‐50 kg at sexual maturity Domestic: 115‐130 kg at sexual maturity (5‐6 mo)	Ewe: <90 kg Ram: <135 kg by 36 mo of age^192^	<90 kg by 36 mo of age
*Behavioral considerations*	Social species with the ability to establish, maintain, or change a relationship. Conflicts within group housing (especially among intact dogs) may result in welfare concerns.	Social hierarchy exists. Aggression and fighting within groups may result in welfare concerns. It is especially recommended intact boars be separated.	Young males are often castrated by 1 mo of age. Calm and docile with relative ease of handling	Goats exhibit distinct behaviors from sheep and they tend to be much more active, curious, and orally investigative.

^a^ The age at skeletal maturity may range depending on the specific breed being utilized.^193^, ^194^ Furthermore, body weight and size of animal can widely vary depending on breed and breeding practices; this is particularly evident in sheep that are bread either for meat or wool (eg, sheep bred for wool are often <50 kg body weight).

^b^ NCD, non‐chondrodystrophic laboratory dogs (eg, Mongrel Hounds); CD, chondrodystrophic laboratory dogs (eg, Beagles). In laboratory animal sciences, the terms “Mongrel dogs” or “laboratory hounds” have become interchangeable in a sense that they refer to non‐Beagle (CD) laboratory dogs. They represent the NCD dog population. These animals are purpose‐bred in USDA Class A dealer facilities which certify clear pedigree and specific pathogen‐free. The benefits of using these animals include avoidance of unclear pedigree and health status which can often be seen in livestock obtained for biomedical research purposes from a sale barn or open herd.

^c^ Max. body weight varies strongly depends on breed.

**FIGURE 1:**
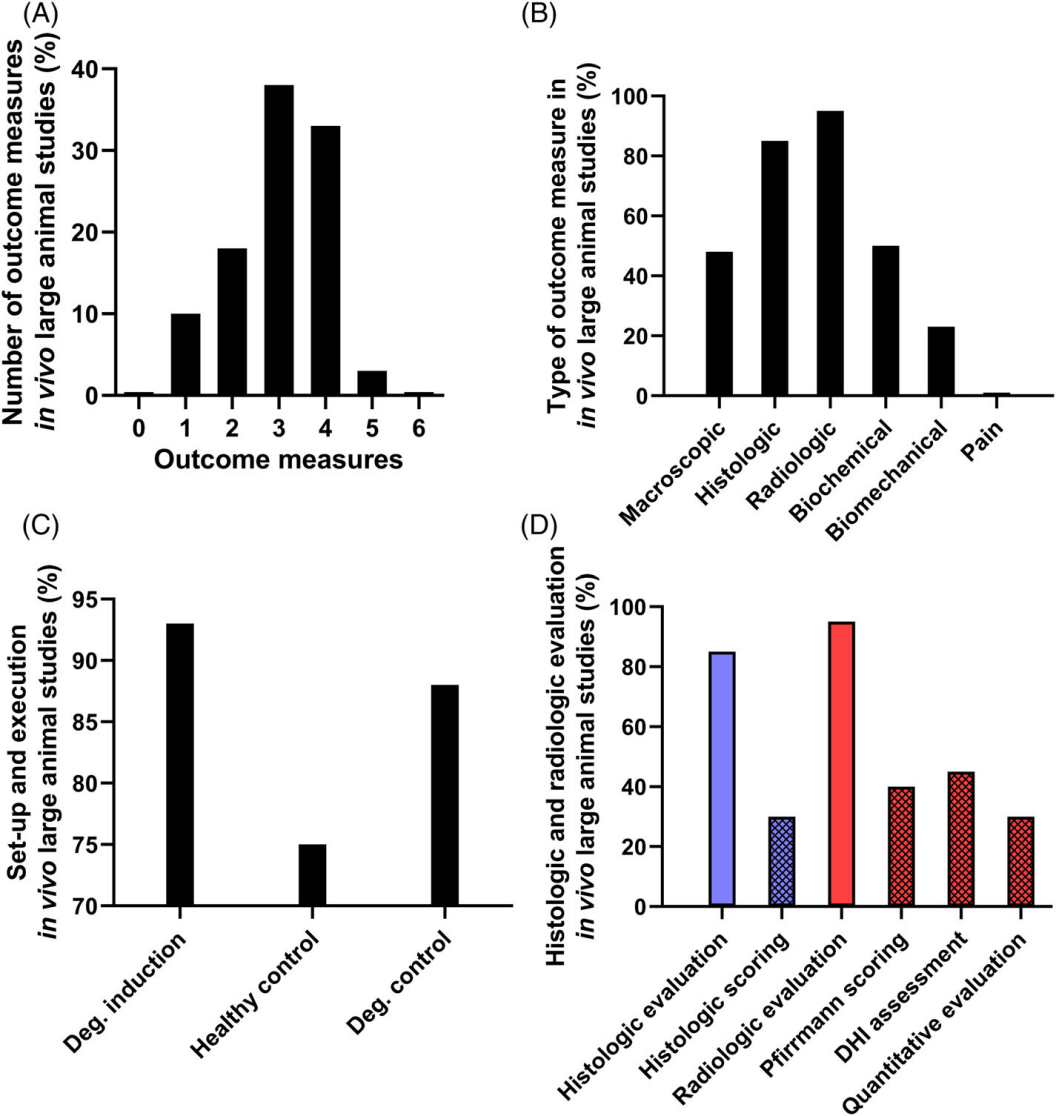
Variety of experimental methodologies reported for outcome evaluation. A sample of recent peer reviewed manuscripts employing four common large animal models (ie, canine, caprine, ovine, porcine; n = 10 per species) to study IVD degeneration or therapeutic strategies in the past two decades. The number of six main outcome measures concomitantly used (macroscopic, histologic, radiologic, biochemical, biomechanical, pain), A, and the detailed use of each type of outcome, B, were registered and demonstrated the majority of the studies employed 3‐4 outcome measures; with limited concomitant biomechanical analysis and absent pain assessment. C, When IVD degeneration was induced (“Deg. Induction”; 92% of all studies), healthy and degenerate controls (“Deg. Control” may be either induced or naturally occurring disc degeneration) were regularly, but not consistently, reported. Spontaneous degeneration was only reported for canine species. Adherence to the ARRIVE guidelines was mentioned in one study. D, Available scoring systems in histological and radiological outcomes (“evaluation” refers to yes/no evaluation and “scoring” specifies within those studies whether or not a scoring scheme was employed), including quantitative MR imaging were seldom employed. The studies included are provided in the Supporting Information, Appendix S1

The large animal model and spinal segments (eg, cervical, lumbar) selected for a particular study are highly dependent on the specific aims of that study. Also, institutions are highly variable in terms of infrastructure supporting animal procurement, housing, biosecurity, access to specialty equipment, and other technical expertise. For example, availability of biomedical research quality‐specific pathogen‐free animal sources is dependent on geographical location and methods in inducing disc degeneration in large animal models are dependent on individual institutions or research groups. There are four species that predominate: dog (canine), goat (caprine), pig (porcine), and sheep (ovine), in alphabetic order. Regardless of the model, the current literature reports a wide variety of experimental methodologies, with three or fewer distinct methodologies employed in most studies (Figure 1; Supporting Information, Appendix S1). This observation was discussed during a breakout session during the ORS PSRS fifth International Spine Research Symposium on large animal models. There was a strong consensus that consolidating these methodologies and their specific application in large animal studies of IVD degeneration and regeneration would represent an invaluable resource for the spine research community. Therefore, by consolidating knowledge from literature as well as expertise, and experiences from the IVD research community, a basic set of tools was compiled allowing for complementary functional and anatomical evaluations in large animal models. Here, a workflow is presented based on general considerations pertaining to study design and tissue processing of spinal specimens, and considerations and suggestions for three scales of read outs: macroscopic (gross morphology, imaging, and biomechanics), microscopic (histological, biochemical, and biomolecular analyses), and clinical (neurologic state, mobility, and pain).

### 1.1 General considerations for study designs

The ARRIVE (Animal Research: Reporting of In Vivo Experiments) guidelines^14^, ^15^ were developed to increase reproducibility and scientific rigor by providing a checklist of information to include when reporting animal studies. Many publishers require manuscripts to closely follow the reporting guidelines and thereby support the 3Rs principles (Replacement, Reduction, and Refinement). To further reduce the number of animals, IVD degeneration can be induced at multiple levels employing nonadjacent levels to limit interference among the IVDs studied. In such a multilevel approach, within each animal/lumbar spine, the following control conditions may be, at a minimum, included for proper comparisons: a nonoperated intact (nondegenerated) IVD, an experimentally degenerated, but untreated IVD, and a sham operated IVD (eg, surgical exposure and vehicle only). The inclusion of these controls may facilitate assessment of the intrinsic repair capacity of the large animal model being employed.

The multilevel approach may complicate understanding the causative relationship between the clinical scale read out parameters (such as painful symptoms) and IVD degeneration or the intervention being studied. In order to remove a confounding factor of having more than one degenerative IVD causing pain and disability, these research questions may require models that involve inducing IVD degeneration at a single level per animal in contrast to the abovementioned multilevel approach.

Longitudinal imaging follows IVDs from their native state to induced degeneration and subsequent experimental therapeutic interventions, thereby allowing determination of changes from baseline measurements. Although the IVDs in each region (lumbar thoracic and cervical) are of relatively similar size, the lumbosacral IVD is larger and subjected to different biomechanical conditions compared to the other lumbar IVDs.^16^Thus, since it is a level where clinical pathologies are commonly found and treated, in both humans and canines, in the authors' opinion the lumbosacral IVD is preferably separated from the others in the experimental design of large animal studies and not pooled or randomized with other levels.

### 1.2 General considerations for processing spinal specimens

**FIGURE 2:**
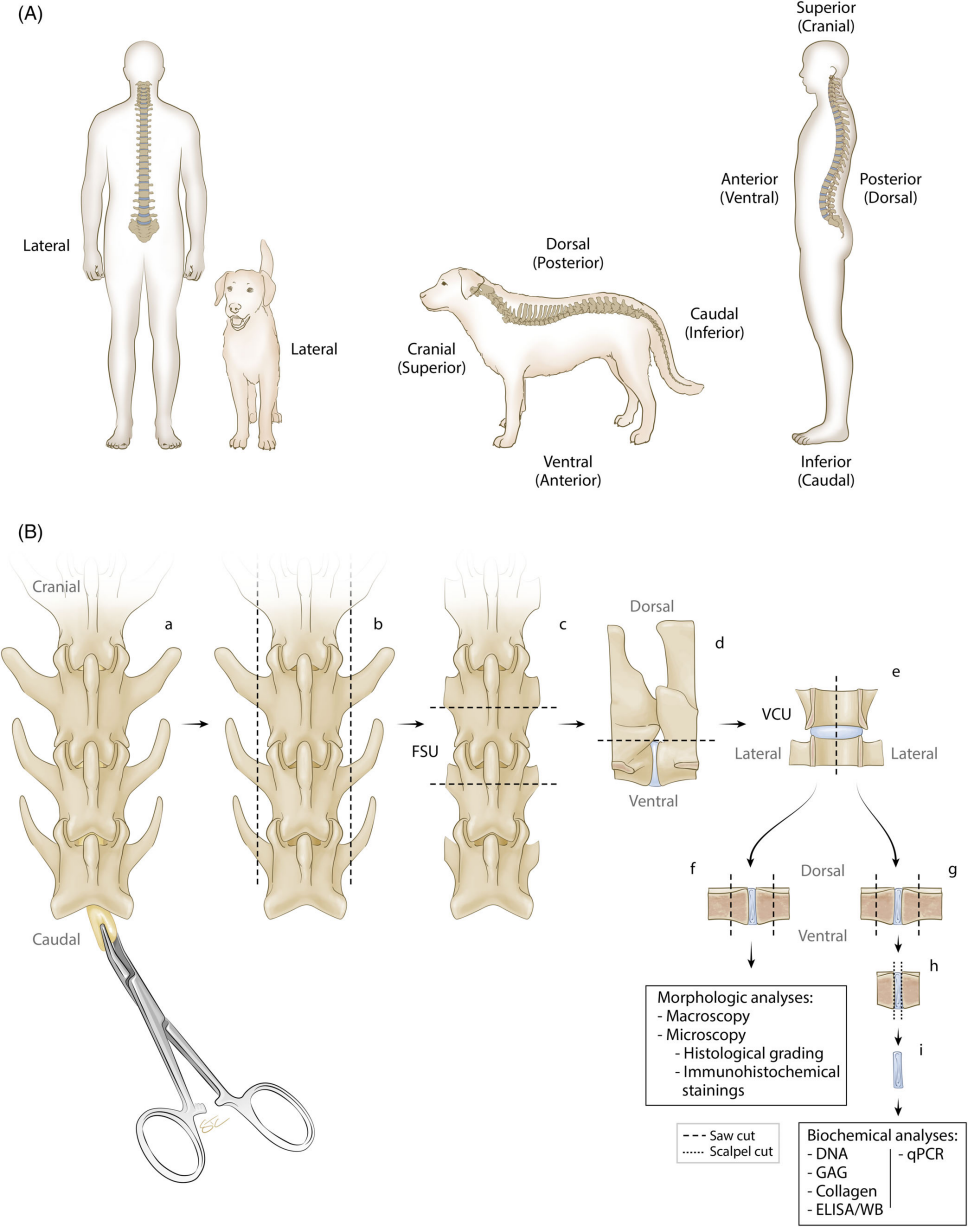
A, Comparative Anatomy of biped and quadruped spines. B, Flowchart of possible *port‐mortem* procedures for evaluation of all read out parameters from each functional spinal unit (FSU). A, Anatomic analogy between humans and quadrupeds. B, Once the spinal column is extracted, the spinal cord can be removed *en bloc* (a). Transverse processes can be removed by trimming the lateral aspects of the spine (b). To isolate FSUs, cuts are made transversely through the mid‐vertebrae (c). Dorsal aspects can be removed by cutting sagittally through the spinal canal (d) resulting in individual ventral column units (VCUs) (e). The isolated VCU can then be transversely transected into two identical parts (e). Thereafter, both parts can be used to take digital photographs for macroscopic evaluation and subsequently, one part may be fixed for histopathology (f). From the second part, the nucleus pulposus (NP) and annulus fibrosus (AF) tissue can be isolated from the cartilaginous end plates (CEPs) and vertebra with a surgical blade for biochemical analysis (g‐i). Note that prior to the described *post‐mortem* procedures (advanced) imaging and non‐destructive biomechanical analysis can be conducted as described in this manuscript

The standard directional anatomical nomenclature for quadrupeds is shown in Figure 2A. By following the process described in Figure 2B, the macro‐ and microscopic parameters described here can be applied to each investigated IVD. As such, gross morphological grading can be combined with histopathological schemes that evaluate cellular and matrix microstructure and molecular compositional information. For all three scales, previously published, well‐validated schemes to evaluate these parameters can be applied and possible drawbacks and opportunities are discussed in the future perspectives section.

Immediately after euthanasia, imaging (eg, plain radiography, computer tomography (CT) and magnetic resonance imaging (MRI)) can be performed in situ, prior to recovering the vertebral column from the animal and extracting the functional spinal units. A functional spinal unit (FSU, also referred to as spinal motion segment [SMS]) comprises one IVD between the adjacent vertebral bodies, intact interbody ligaments, and facet joints, but with paraspinal muscle tissue removed. The pedicles and posterior column elements are typically removed to isolate the ventral column unit (VCU, equivalent to anterior column unit (ACU) in humans; Figure 2B). Isolated VCUs can be further divided transversely (axially) or sagittally for macroscopic evaluation and further processing. Transversely cutting the IVD will result in a cross‐section consisting of the AF and NP. This cut is acceptable for studies that focus on basic analyses of AF and NP; however, evaluation of the cartilaginous endplate (CEP) is not possible. Sagittal sectioning, which preserves the anatomic orientation and context between components of the FSU, is suggested for macroscopic and microscopical evaluation and grading of AF, NP, and CEP as shown in Figure 2B. The workflow proposed by the authors in Figure 2B and the Supporting Information (Appendix S1) allows for the complete evaluation of gross morphological analysis in combination with imaging, histology, biochemical, and biomechanical analysis (outlined in more detail in the Supplementary flowchart, Appendix S1). Transverse sectioning prior to fixation is not recommended as without constraint of the IVD by the vertebral bodies, the NP tissue may swell, distorting tissue architecture and allowing leaching out of extracellular matrix (ECM) components during subsequent tissue processing.^17^ Instead, *en bloc* fixation of the intact VCU can prevent these artifacts.

Combining multiple types of characterizations of tissue samples provides an in‐depth understanding of IVD regeneration. Proper planning is necessary so that nondestructive procedures, like imaging and biomechanics, can be carried out prior to destructive tests such as gross morphological evaluation, or those that alter the physical properties of the tissue, such as fixation for histology. If storage is unavoidable, a single freeze‐thaw cycle is generally considered acceptable prior to imaging or biomechanics, with the caveat that this may partially compromise planned macro‐ and microscale outcome parameters. Preparation of tissue for RNA or protein assays is preferably done using freshly isolated, unfixed, and undecalcified specimens. For biochemical and biomolecular analysis, tissue is usually snap frozen in liquid nitrogen and stored at −80°C.^18^ For biomechanical testing, the use of fresh specimens is preferred, but if this is not feasible, specimens can be properly stored in controlled conditions until testing as described in the respective section.

### 1.3 Macroscopic scale

#### 1.3.1 Gross morphological grading

Gross morphological grading refers to the scoring of an IVD's pathological state through inspection of internal macrostructure, either directly or using photographs. Direct physical inspection, while posing some practical limitations, has advantages over digital imaging, as tactile assessment of the exposed tissue enables more effective localization of defects such as AF delamination and circumferential tears, rim lesions, and NP tissue granulation. Nevertheless, high‐quality photographs are most frequently used for macroscopic evaluation.

The most commonly adopted gross morphological grading scheme for IVDs was published by Thompson et al.^19^ This scheme is based on a refinement of the comprehensive descriptions of human IVD pathology by Vernon‐Roberts,^20^ and includes assessments of the major substructures of the IVD (NP, AF, and CEPs) in addition to the adjacent vertebral body margins. In developing and validating this scheme, Thompson et al applied it to 68 IVDs from 15 human spines obtained *postmortem* from individuals aged 16‐89 years. Mid‐sagittal VCU sections (including the osseous endplates) were graded by three experienced and blinded individuals, and the authors undertook extensive validation to ascertain intra‐ and interoperator variability, which were found to be 85%‐87%, and 61%‐88%, respectively.

**FIGURE 3:**
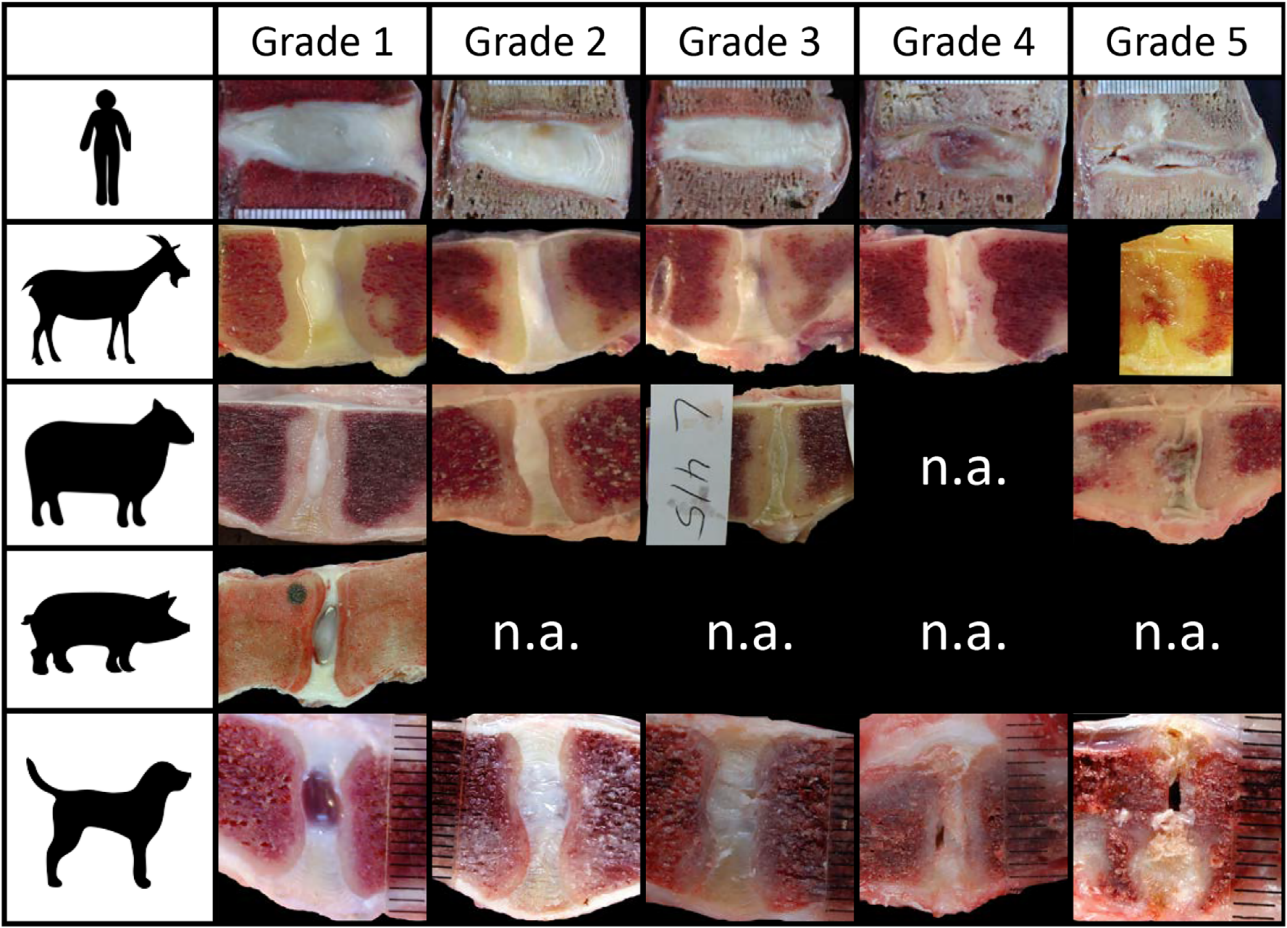
Gross Morphology. Exemplary gross morphological grading images for discs from goats (caprine), sheep (ovine), pigs (porcine) and dogs (canine). Human IVDs are included for comparison. Grades (1–5) have been assigned according to the criteria outlined by Thompson et al,^19^ where Grade 1 corresponds to healthy IVDs and Grade 5 the most severely degenerated. Noteworthy characteristics specific to each image series include: the translucent, notochordal NP region present in healthy dog and pig IVDs, compared to the more cartilaginous NP in healthy sheep, goat and human IVDs; and the presence of growth plates in sexual and skeletal maturity goat and sheep and in the immature pigs. Gross morphological grading includes assessments of the major substructures of the IVD (NP, AF, and CEPs) in addition to the adjacent vertebral body margins and as such, the gross changes may differ depending on the method of disc degeneration and the therapeutic approach tested. Examples in this figure are from induced disc degeneration models via chondroitinase ABC (goat) and partial nuclectomy (ovine) and from naturally occurring disc degeneration (canine). The worst grade of the different substructures is used to define the final score. Animal IVDs are oriented with the ventral side facing down. Human IVDs are oriented with the anterior side facing to the right and are from naturally occurring disc degeneration. n.a.: not available; Relative size differences need to be considered between the different species; grading is independent of the relative IVD size. Goat, dog, and pig images were kindly provided by Prof. Dr. Theo Smit, Dr. Niklas Bergknut, Prof. Dr. Hans‐Joachim Wilke respectively. Images of human IVDs were adapted from Wilke et al^198^ and Galbusera et al^199^

While this scheme was originally designed for application to human IVDs (giving it high clinical relevance), it has frequently been adapted for large animal IVDs. A literature search revealed approximately 60 studies that cite the Thompson grading scheme with application to dog,^21^ sheep,^22^ pig,^23^ or goat IVDs^24^ (select examples cited with examples for each species shown in Figure 3). Of note, the Thompson grading scheme has been adapted and applied to IVDs cut in the transverse plane by some researchers^25^; however, this presents the significant limitation that the CEPs, BEPs, and vertebral bodies cannot be assessed. In instances where this, and other significant deviations from the original scheme are implemented, additional assessment of inter‐ and intraoperator variability may be warranted.

In reflecting whether to employ gross morphological grading, the following can be considered: (a) the ease of integrating macroscopic evaluation into the overall experiment workflow; (b) available access to at least two independent evaluators (experts or trainable personnel) to perform accurate and consistent evaluations of IVD morphology; and (c) the relative advantages and disadvantages that macroscopic evaluation provides over other types of assessments, if all cannot be performed. With respect to this final consideration, advantages of gross morphological grading include its low cost, the requirement for minimal specialized equipment or technical expertise, and its ability to provide a higher resolution assessment of IVD and vertebral bone macrostructure compared to radiologic/MRI evaluations. Unlike histology, there is minimal pre‐analytical variability that could impact results (eg, tissue microtomy artifacts due to tissue shrinking or expansion, damage from sub‐sectioning, or variations in staining). Accurate and consistent histologic evaluation typically requires specialized training so that tissue artifacts listed above are not misinterpreted as actual lesions (eg, tears in the AF matrix degeneration). The major disadvantages of macroscopic evaluations include acquiring subsections that result in tissue destruction, and unlike imaging, it cannot be applied longitudinally *in vivo*. Macroscopic evaluation schemes are also inherently subjective, with grades potentially subject to ambiguity and high interobserver variability even when performed by experts.

#### 1.3.2 Implementation

The parameters described herein for gross morphological grading of large animal IVDs are drawn in large part from the original study of Thompson et al^19^ (to which readers are encouraged to refer). IVDs should be bisected using a single, smooth cut using a straight‐edged trimming knife or similar implement, in order to minimize cutting artifact that could confound assessments. Following the scheme of Thompson et al,^19^ IVDs are assigned a grade from 1 to 5, where 1 is healthy and 5 is most degenerate. Grading criteria for each IVD substructure (NP, AF, CEP, and vertebral bodies) are shown in Table 2 (example images in Figure 3). Both left and right hemi‐IVDs should be graded and averaged for each of the assessors.

**TABLE 2:** The macroscopic grading scheme adapted from Thompson et al^19^

Grade	Nucleus pulposus (NP)	Annulus fibrosus (AF)	Cartilaginous end plates	Vertebral bodies
1	Bulging gel	Discrete fibrous lamellae	Hyaline; uniform thickness	Rounded margins
2	White fibrous tissue peripherally	Mucinous material between lamellae in inner AF	Irregular thickness	Pointed margins
3	Consolidated fibrous tissue	Extensive mucinous infiltration; loss of annular‐nuclear demarcation, in‐folding of inner AF lamellae	Focal defects in cartilage	Early chondrophytes or osteophytes at margins
4	Horizontal (vertical) clefts parallel to end plate	Focal disruptions	Fibrocartilage extending from subchondral bone; irregularity and focal sclerosis in subchondral bone	Osteophytes <2 mm
5	Clefts extend through NP and AF		Diffuse sclerosis	Osteophytes >2 mm

### 1.4 Imaging

Imaging is frequently employed for the evaluation of IVD degeneration/regeneration in experimental large animal models, noninvasive longitudinal assessments during in vivo studies. Imaging is also commonly performed on ex vivo samples at study end points. The most common imaging modalities applied are plain radiography and MRI, while other advanced imaging modalities may be available in specific research infrastructures, including computed tomography (CT) and ultra‐high field MRI, providing additional spatial information with improved resolution.

#### 1.4.1 Plain radiography

Plain radiography is a widely used and accessible tool to evaluate the boney structures of the spine. Depending on the model and the research approach, radiographs can provide information on morphology and density of vertebral bodies, IVD degenerative changes including IVD height loss, spondylolisthesis,^26^ osteophyte formation, or bridging via spondylosis formation in late stages of IVD degeneration. Additionally, information on possible adverse effects of the therapeutic approach can be indicated by radiographs, for example, extradiscal bone formation following intradiscal injections due to leakage of the therapeutic agent. Compared with other imaging techniques, some advantages of plain radiographs are the low cost and high availability across institutions.

Depending on the infrastructure and equipment, radiographs can be obtained either in the standing position (goat, sheep) or conducted under deep sedation or anesthesia (dog, pig, sheep, goat) to ensure proper muscle relaxation, and to allow for positioning and minimizing distress to the animal according to “Refinement,” one of the 3Rs principles. Positioning of the animals for radiographic evaluation depends on the spinal segment of interest; ideally the region of interest is centered in the radiograph as parallax errors increase at the borders of the image. Furthermore, it is important to maintain the vertebral column parallel and as close as possible to the x‐ray cassette for a better evaluation of IVD spaces. General aspects to further consider are tight collimation to enhance detail. Next to the most common lateral view, ancillary views such as ventrodorsal, flexed, and extended views can be acquired when evaluating cervical IVDs.^27^

**FIGURE 4:**
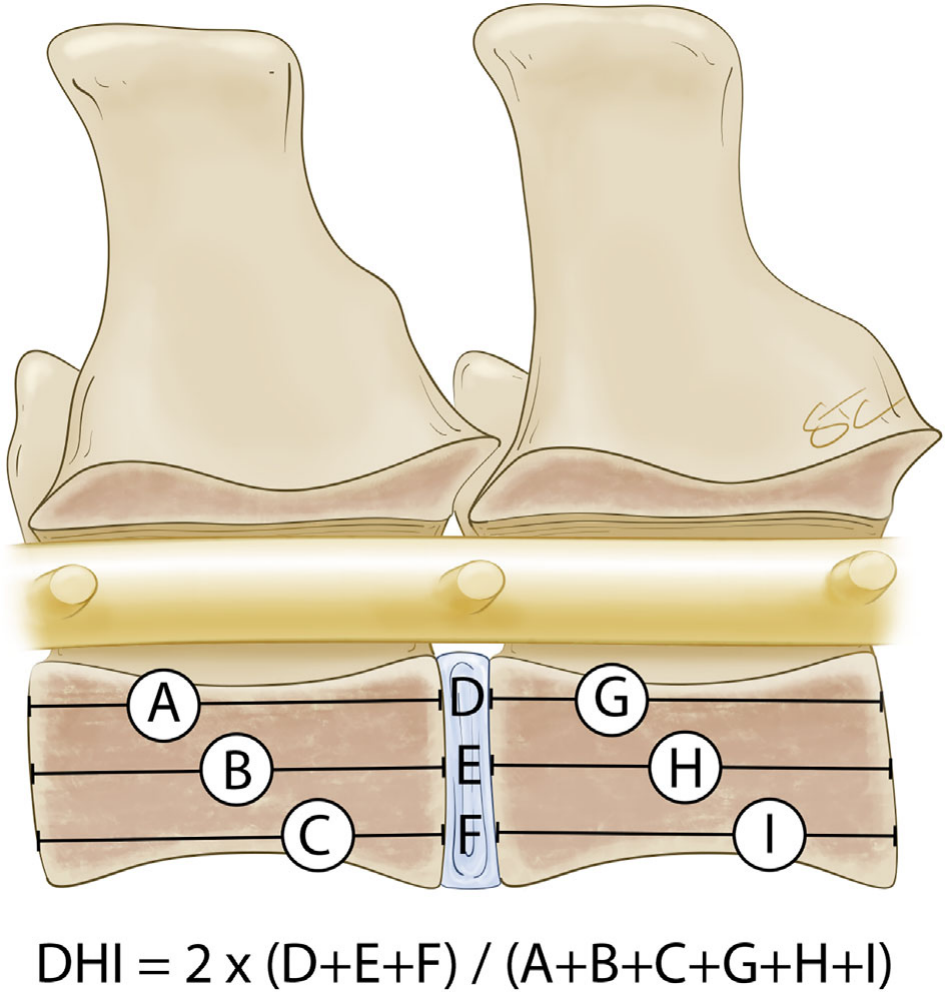
The disc height index. It is suggested to quantify changes in IVD height on plain radiographs or high field MR images. According to Masuda et al^28^

Disc height index (DHI) quantifies changes in IVD height (Figure 4) and is considered a more accurate method than absolute IVD height measurement as it corrects for positioning and animals of differing sizes. The DHI is calculated by taking the average of two‐dimensional measurements obtained from the dorsal, middle, and ventral portions of the IVD and dividing those by the average of the adjacent vertebral body heights.^28^ The DHI was proposed by Sasaki et al^29^ and thoroughly studied and advanced by Masuda et al.^30^ It is essential to maintain a consistent level of sedation (or no sedation) during radiography of each animal and at each time point, in order to obtain a similar degree of muscle relaxation and minimize confounding effects on IVD space narrowing. These measurements are most frequently obtained using plain radiographs; although the resolution and three‐dimensionality afforded by MRI may allow for more accurate measurements.

#### 1.4.2 Magnetic resonance imaging

Currently, MRI is considered the most definitive noninvasive imaging tool in the clinical assessment of IVD degeneration, and in large animal models is always conducted under anesthesia to prevent motion artifacts. Within this context, little is known on how the level of relaxation in large animal models may affect outcomes, likewise diurnal variations on signal intensity and IVD height reported in humans.^31^ In terms of grading degeneration, MRI provides valuable information on signal intensity (related to hydration state), IVD height and the structure and differentiation of its components. Complications such as extradiscal bone formation due to leakage of stem cells or growth factors,^32^, ^33^ or other lesions including Schmorls nodes,^34^ can also be detected by MRI.

**FIGURE 5:**
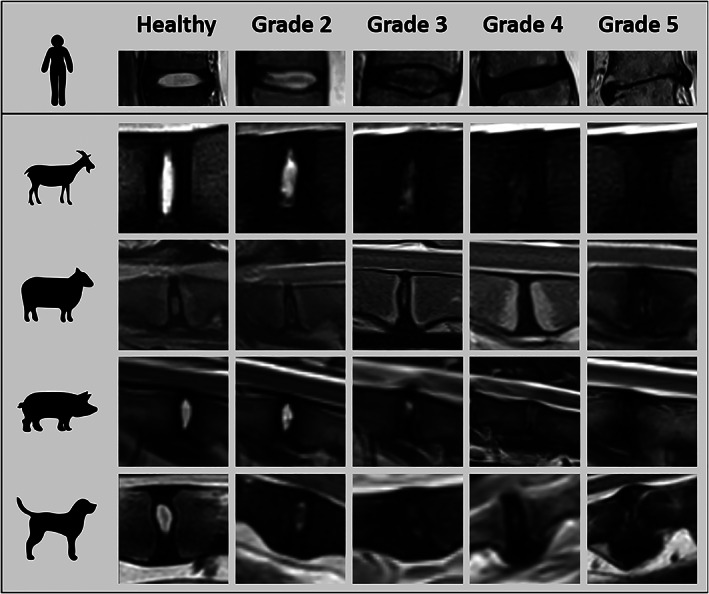
Healthy and degenerated IVDs (Pfirrmann graded) in large animal species and human. Typical examples of T2‐weighted MR images from human (3 T); goat, sheep and pig with experimentally induced IVD degeneration (3 T), and dog with naturally occurring IVD degeneration (1.5 T). Note that the intensity of the IVD signal is compared to the intensity of the cerebrospinal fluid (Table 6) Grade 1: The healthy IVD shows a homogenous structure with a hyperintense signal intensity and normal IVD height. Grade 2: The structure of the IVD is no longer homogeneous and the signal is still intense. Horizontal gray bands may be present in the IVD that is related to a beginning unclear distinction between NP and AF. Grade 3: Signal intensity is intermediate and the height of the IVD is slightly but visibly decreased with unclear distinction between NP and AF. Grade 4: Signal intensity is hypointense and there is no longer a distinction between NP and AF, the IVD height is moderately decreased. Grade 5: Inhomogeneous structure of the IVD with a hypointense signal intensity and a collapsed IVD space. Note that in naturally occurring IVD disease at these stages spondylosis occurs eventually, potentially fusing the segment with progression (eg, dog, Grade 5). NP: nucleus pulposus, AF: annulus fibrosus. Human and sheep MRIs were kindly provided by Frank Niemeyer^169^ and Marion Fussilier,^178^ respectively

In 2001, a grading system using MRI was developed by Pfirrmann et al,^35^ for the semiquantitative assessment of the human lumbar IVD degeneration condition. T2‐weighted‐MRI sequences without fat saturation were used for this purpose as the signal loss of the IVD on these sequences correlates with progressive degenerative changes. The Pfirrmann scale ranges from 1 to 5 and considers: the structure and signal intensity of the NP, the height of the IVD, as well as the distinction of the NP and AF (Table 3, Figure 5). This grading system is still the most accepted and commonly used MRI grading system for the evaluation of IVD degeneration.^36^, ^37^ The Pfirrmann grading system was proposed using 1 T MRI images almost 20 years ago, and the quality of images of high and low magnetic fields in MRI have improved since then. As such, an updated MRI disc degeneration grading system may be considered.^38^ For this purpose, the present manuscript provides representative images for the four commonly used large animal species in the field (Figure 5).

**TABLE 3:** The MRI‐based grading scheme adapted from Pfirrmann et al^35^

Grade	Structure	Distinction between NP and AF	Signal intensity	Height of IVD
1	Homogeneous and bright white	Clear	Hyperintense and isointense to CSF	Normal
2	Nonhomogeneous with or without horizontal bands	Clear	Hyperintense and isointense to CSF	Normal
3	Nonhomogeneous and gray	Unclear	Intermediate	Normal to slightly decreased
4	Nonhomogeneous and gray to black	Lost	Intermediate to hypointense	Normal to moderately decreased
5	Nonhomogeneous and black	Lost	Hypointense	Collapsed disc space

*Notes*: Pfirrmann grading is performed using T2‐weighted sequences with 1.5 Tesla or higher. From the high‐quality images, four factors are scored: overall structure, the distinction of specific components, signal intensity, and the height of the IVD.

Abbreviations: AF, Annulus fibrosus; CSF, cerebrospinal fluid; NP, Nucleus pulposus.

Various quantitative MR sequences have been shown to reliably indicate biochemical changes in the IVD, including hydration status, proteoglycan content, and IVD degeneration as reported for goats,^39^ sheep,^40^ pigs,^41^ and dogs.^42^ Relying on signal intensity alone (eg, from single T2‐weighted images) for quantitative assessments is problematic, as signal intensity will vary based on position in the magnet. In the absence of performing truly quantitative MR sequences, normalizing IVD signal intensity to that of adjacent structures such as cerebral spinal fluid or bone marrow (which are expected to remain constant) is one option to address this. Considering the advantages of qualitative and quantitative MRI, researchers can plan longitudinal follow up during in vivo studies. Ideally, MRI analysis is conducted prior to induction of IVD degeneration and once degeneration is established prior to the administration of therapies. This not only allows determination of baseline measurements, but also enables registration of confounders that may affect interpretation of the study results, such as variability in the level of IVD degeneration and pathologic changes, like the less documented vertebral body and endplate changes termed “Modic changes” at the vertebral body level.^43^

### 1.5 Biomechanical assessment

**FIGURE 6:**
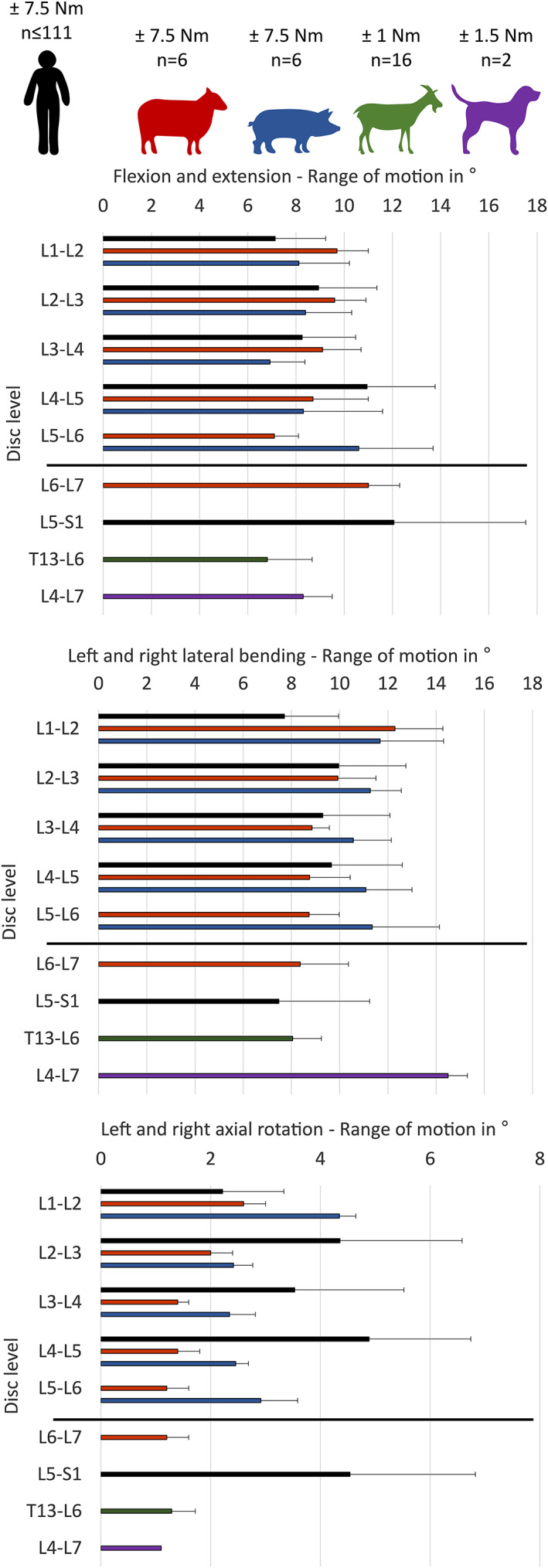
The range of motion (ROM) of healthy lumbar IVDs. The ROM of individual healthy lumbar IVD levels of sheep^200^ and pig^201^ as well as the average ROM of dog (average from L4‐L5 to L6‐L7, unpublished preliminary data from Prof. Dr Hans‐Joachim Wilke) and goat^202^ (average from T13‐L1 to L5‐L6) compared to human^203^ specimens (unpublished ROM data, described in Kettler et al^203^). Note that the sheep spine contains 6 or 7, the dog 7, the goat and the pig 6 lumbar vertebrae compared to 5 in human. Values are mean (SD) ROM in ° for each species; the moment and number of IVDs studied for the large animal models is given in the figure caption. Pig: 15 spines from 6‐month‐old cross of Pie'train boar with hybrid pig. Sheep: 14 spines from 3 to 4‐year‐old female merino sheep. Goat: 8 spine from 3‐ to 4‐year‐old female Dutch milk goats. Dog: 2 spines from ~2 years old non‐chondrodystrophic dogs. Human: 111 adult donors

The IVDs of large quadrupedal animals are often assumed to be under different loads than those of humans because of the difference in horizontal vs vertical spine orientation, along with other anatomical differences. However, to keep the spine of quadrupeds in a horizontal position, substantial contractile forces arising from combinations of muscular contraction and reactive forces in the ligaments are necessary, as evidenced by the density and orientation of their vertebral trabecular structure.^10^, ^44^, ^45^ As such, the basic biomechanics of large animal spines are not much different to those of humans, and therefore, changes in biomechanical properties evaluated in these models have translational applicability to human IVD health and disease. Nevertheless, the degeneration model employed may interfere with the evaluation of the biomechanical effects of the treatment strategy, such as cavity formation by enzymatic digestion.^46^ A basic description of IVD biomechanics and load displacement curves (LDC) is provided in the Supporting Information (Appendix S1) and the normal, healthy lumbar range of motion (ROM) for the common experimental large animal species in comparison to those of humans is shown in Figure 6.

The most common spine sample type for biomechanical evaluation is the FSU, as it is the basic functional repeating unit of the spine; however, two or more coupled FSUs are also sometimes evaluated. To investigate the biomechanical properties of only the IVD, VCUs are often employed. Although their biomechanical behaviors are related, different levels of FSUs or VCUs are ideally compared individually.^47^, ^48^ If the research question and the infrastructure allow, nondestructive biomechanical testing on freshly isolated specimens which can be further processed for complementary readouts is ideal (Figure 2; Supporting Information, Appendix S1).

### 1.6 Ex vivo biomechanical testing

#### 1.6.1 Specimen preparation and storage

Whenever possible, fresh specimens are preferred for biomechanical testing. If specimens need to be stored, a 0.15 M PBS‐soaked gauze can be placed around the unit and subsequently covered by at least two plastic bags/cling foils^49^ to prevent dehydration. Generally, mainly cartilaginous specimens (ie, IVDs not containing NP notochordal cells) may be stored at 4°C for at most overnight without a negative impact.^50^ For a longer duration, specimens may be frozen, at −20°C or lower for best sample preservation based on the available literature body which is summarized in detail in the Supporting Information (Appendix S1). For the retrieval and storage of IVD tissues (CEP, NP, and AF), we suggest storing the entire intact IVD and harvesting the tissue directly before testing, if the tissue cannot be tested fresh.^51^

#### 1.6.2 Test preparations

Prior to testing, the tissue or IVD specimen can be thawed while sealed to prevent dehydration. If samples need to be tested multiple times over consecutive days, freeze‐thawing can be avoided by storing at 4°C and sealing to prevent dehydration.^52^ The loading history of the sample influences the reproducibility of the biomechanical evaluations, for example, due to variations in hydration.^53^, ^54^, ^55^ The IVD height varies during day and night (diurnal changes) due to water flux caused by the changing average load magnitude. This affects, among other parameters, the intradiscal pressure.^56^ There may be water content variations from storage conditions, which are ideally balanced out prior to testing. This can be addressed by applying axial compression load (“pre‐load”) or displacement, or allow passive hydration without load with the goal to equilibrate to a physiologically realistic situation.^57^

During testing, the specimens are frequently kept in a temperature and humidity‐controlled test environment that best mimics the in vivo situation. Accordingly, temperature is kept constant at 37°C, or alternatively room temperature at a relative humidity of ca. 100% at all times. Humidity can be controlled, for example, by spraying 0.15 M PBS, in an environmental chamber, wrapping in soaked gauze or by creating a reservoir.^48^, ^58^ Of note, swelling will occur in a saline bath without axial loading.^59^ At 37°C, catabolic enzymes are active, and degeneration is enhanced. Therefore, specimens cannot be tested for a longer duration than several hours without the use of protease inhibitors and antifungal agents. Biomechanical experiments are frequently carried out at room temperature, however, the viscoelastic properties of the IVD and adjacent structures are temperature dependent.^60^, ^61^

Specimens are frequently “preconditioned,” usually for at least three cycles to achieve a consistent response in creep,^62^ stress relaxation, and in load‐displacement curves.^47^ Furthermore, the strain rate, preload, and follower load can be optimized in preliminary studies, as they are, for example, dependent on animal, IVD size and level, age, biomechanical test, or degeneration state.

#### 1.6.3 Testing of FSU or VCU specimens

For multiple degrees‐of‐freedom (see Supporting Information, Appendix S1) FSU‐testing and analysis recommendations, we refer to the review by Wilke et al.^47^ In addition, individual VCU specimens may be tested in unconfined compression^63^ to measure axial, time‐dependent characteristics, for example, creep‐recovery.^49^, ^64^ Biomechanical parameters of healthy IVDs are IVD‐level dependent and as such should not be averaged on a spine region but evaluated individually.^65^

#### 1.6.4 Testing the AF, NP, and CEP

The AF lamellae experience high circumferential and longitudinal tensile, shear, and radial compression forces. The orientation during testing is relevant as the tissue is anisotropic.^66^ The AF can be tested in uni‐ or biaxial tension, in shear, or under unconfined or confined compression (ie, with more controlled boundary conditions). For each test, single or multiple lamellae may be used.^48^, ^67^, ^68^

Due to a high fixed charge density, the NP can imbibe large amounts of water. If the glycosaminoglycans (GAGs) responsible for this swelling and the water content are altered (because GAGs leach out or water is imbibed in unloaded conditions^69^), the time‐dependent mechanical properties change drastically.^70^, ^71^, ^72^ Furthermore, the NP experiences shear stresses, for example, during bending or axial rotation. As the NP is in vivo confined by the bony and cartilaginous endplates (BEP and CEP) and the AF, compression and relaxation tests are frequently performed in confined conditions.^73^, ^74^ Unconfined compression is not physiological, but may be used to determine NP material parameters, for example, Poisson's ratio.^75^ During axial loading, the intradiscal pressure increases in the confined NP, which can be measured.^76^, ^77^ Reitmaier et al have published a method for in vivo measurements of intradiscal pressures in large animals^78^ and have compared this to human intradiscal pressures.^79^

Compared to the NP and AF, little attention has been given to the CEP, even though it plays an important role in IVD degeneration.^80^ Inhibition of endplate nutritional pathways leads to IVD degeneration^81^ and the CEP it is also responsible for limiting the loss of NP matrix proteins. Thus, CEP properties should ideally be considered when studying IVD regeneration, especially because solute transport might be affected, for example, due to CEP degradation caused by enzymatically induced IVD degeneration. Biomechanical evaluation of the CEP can include confined compression^82^ or uniaxial tension,^83^ or permeability testing^84^, ^85^testing.

### 1.7 Microscopic scale

The overall goal of microscale evaluation is to provide characterization of the microscopic architecture and composition of the IVD, including cellular and ECM components at the histological, biochemical, and biomolecular level.

#### 1.7.1 Histopathology

For histological grading, it is standard practice to evaluate the microstructure of the NP, AF, and CEP on mid‐sagittal tissue sections; however, if a region of interest of the IVD relevant to a specific treatment type or pathology is to be examined, the direction of sectioning should be adapted accordingly. For example, if histologic evaluation of the endplate is not needed, transverse sections can be used to provide more surface area of NP and AF to evaluate (personal communication, Dr Chantelle Bozynski, Missouri Orthopedic Institute). A properly prepared section is essential to accurately observe the fine cellular and tissue component structures. Here, we describe common fixation and processing procedures, and scoring systems for microscale analyses, providing technical and analytical considerations, and potential drawbacks with respect to their application. A more detailed version of this histopathology section is provided in the Supporting Information (Appendix S1).

#### 1.7.2 Tissue processing for histopathology

##### Fixation

Fixation and decalcification procedures are very important for proper histological analysis of IVDs. Although cryo‐compound fixation and cryo‐sectioning can be performed for IVD analysis, in the authors' experience, stained sections of paraffin embedded decalcified specimens provide superior morphology for evaluation of cellular and ECM components. However, histologic tissue artifacts that might unintentionally be generated during fixation, decalcification, dehydration, or sectioning procedures should not be confused with authentic degenerative features in IVD tissues (see Table 4). For most applications, IVD tissues are fixed to preserve the cells and ECM and prevent autolysis. To minimize the required fixation time and possible confounding effects of inappropriate fixation (eg, leaching out of ECM molecules^17^), the sample can be trimmed to the required size before fixation (Figure 2B). To prevent unconstrained swelling of the NP tissue during fixation, the CEP needs to be maintained. Frequently used fixatives for IVD research purposes are 10% neutral buffered formalin or 4% paraformaldehyde. Cryo‐compound fixation of samples immediately after removal of the CEPs can allow for fast processing with postfixing after sectioning.^86^, ^87^, ^88^ For immunohistochemistry, ethanol fixation can avoid some of the antigen retrieval steps.

**TABLE 4:** Histological grading scheme of disc degeneration compiled based on validated schemes^40^, ^96^, ^97^, ^98^, ^99^, ^100^

of notochordal cells (NCs)^a^ in the NP (H & E; dog and pig model only)—Figure 9	
Abundantly present (>90% of total cells) within the entire NP	0
Present in moderate amounts (50%‐90% of total cells)	1
Present in low amounts (<50% of total cells)	2
Absent	3

Nucleus pulposus cell (NPC)^b^ clusters (H&E)—Figure 10 and 11	
No clusters	0
Scattered small cell clusters (ie, 2‐7 nuclei per cluster)	1
Frequent large cell clusters (ie, >8 nuclei per cluster)	2
Presence of huge cell clusters (ie, >15 nuclei per cluster)	3

NP cell loss (karyolysis) and cell necrosis (pyknosis or karyorrhexis) (H&E)—Figure 11 and Figure 12	
None—lacunae all contain viable nuclei	0
Rarely present. Few (<25%) lacunae demonstrate karyolysis, pyknosis, or karyorrhexis	1
Frequently present. 25%‐50% of lacunae demonstrate karyolysis, pyknosis, or karyorrhexis	2
Karyolysis, pyknosis, or karyorrhexis predominates (>50%)	3

NP Matrix staining (AB‐PSR)—Figures 7 and 10	
Blue proteoglycan ECM stain dominates	0
Reduction in blue proteoglycan ECM staining (ie, fading)	1
Reduction in blue proteoglycan ECM staining with presence of collagen staining (up to 50% of area)	2
Loss of blue proteoglycan ECM staining and/or dominance of collagen staining (>50% of area)	3

AF morphology (AB‐PSR)—Figure 13	
Well‐organized, well‐defined, uniform collagen lamellae form concentric half‐ring arcs throughout entire AF	0
Mild disorganization/delamination of collagen fiber lamellae with some disruption or loss of concentric layers (<25%)	1
Moderately disorganization/delamination of collagen fiber lamellae with progressive disruption or loss of concentric layer (25‐75%)	2
Complete disorganization/delamination/collapse of AF; almost all concentric collagen lamellae are severely disrupted or lost (>75%)	3

AF Cellular (H&E) and ECM metaplasia^c^ (AB‐PSR)/distinction between AF and NP—Figure 14 and Figure 15	
Clear distinction between AF and NP tissue with intense blue proteoglycan ECM staining in NP: spindle‐shaped fibrocytic nuclei populate AF with no or rare individual metaplastic cells resembling NPCs in the AF	0
Distinction less clear: loss of annular‐nuclear demarcation. Proliferation of cells resembling NPC's is focally restricted (ie, perilesional) or mild proliferation with >75% lacunae containing single fibrocytic nuclei (ie, rare to few small NPC‐like clusters)	1
Distinction less clear: loss of annular‐nuclear demarcation. Regionally extensive perilesional proliferation of cells resembling NPC's or moderate proliferation involving 25%‐50% AF lacunae that contain more than one cell nucleus (ie, frequent NPC‐like clusters).	2
Poorly or no discernable annular‐nuclear demarcation. Regionally extensive perilesional proliferation of cells resembling NPC's or moderate proliferation involving >50% AF lacunae that contain more than one cell nucleus (ie, frequent NPC‐like clusters).	3

Tears and cleft formation^d^ in the AF/NP (H&E, AB‐PSR)—Figure 13 and Figure 15; Figure 18	
Absent	0
Rarely present. Few small concentric‐oriented clefts confined within a single region of the AF (ie, inner, mid or outer layer), without formation of transverse clefts	1
Larger and more numerous clefts or formation of transverse clefts that bridge an adjacent AF region (ie, inner‐to‐mid, or outer‐to‐mid)	2
Abundantly present, large clefts; formation of transverse clefts that bridge more than one layer of the AF; or presence of cellular infiltration/neovascularization	3

CEP morphology (Saf O/FG, AB‐PSR)—Figures 16, 17, and 18	
Uniformly thickness and contour without disruption	0
Focal to multifocal thickness and/or contour irregularities without disruption	1
Focal endplate disruptions (thin clefts, fissures or chondroid ECM extrusion up to 10% total area with or without irregularities in thickness/contour	2
Frequent or large areas of endplate disruption with chondroid ECM extrusion >10% total area with or without formation of Schmorl's nodes	3

Bone modeling—at the external AF/bone interface (H&E)—Figures 7, 8, and 19	
Absent	0
Limited, bone remodeling that forms pointed EP contours or small nodular exostoses that to not impinge on AF	1
Larger vertebral exostoses that protrude cranio‐caudally and impinge on outer layers of the AF	2
Abundant new bone formation with partial to complete bridging spondylosis	3

*Notes*: All categories apply to all species reviewed herein; however, modifications of the scheme or analysis protocols (eg, additional histochemical or immunohistochemical stains) may be warranted, depending on the induction method employed to create a lesion (eg, surgical AF disruption vs chemical‐induced nucleotomy) or the research question (eg, for processes concerning angiogenesis, nerve ingrowth and inflammation, repair strategy employed). The reader is referred to Figures 7, 8, 9, 10, 11, 12, 13, 14, 15, 16, 17, 18 for representative bright field microscopy examples of the different scoring categories. It remains to be determined when reactive degenerative changes included in the scoring may reflect a regenerative response in the context of a regenerative therapy.

Abbreviations: AB‐PSR, Alcain blue/picrosirius red stain; AF, annulus fibrosus; CEP, cartilaginous endplate; ECM, Extracellular matrix; H&E, hematoxylin & eosin stain; NP, nucleus pulposus; NPC, NP cells; Saf O/FG, safranin O/fast green stain.

^a^ Notochordal cells are large vacuolated cells present within embryonic development in the notochord and once the IVD has developed, reside within the NP. There are slight differences in the morphology of the NCs between species (pigs, non‐chondrodystrophic dogs; Figure S5), whereas some species (sheep, goat) do not have vacuolated NCs at the ages they are used in animal experiments therefore this component of the grading scheme should not be applied to those models. The non‐vacuolated smaller NP cells residing within the core of the IVD are termed NP cells (NPCs).

^b^ Note that NPC clusters as presented in this article are analogous to “chondrocyte proliferation” as presented in other articles (eg, Bergknut et al^97^ and Boos et al^94^).

^c^ Note that cellular and ECM metaplasia presented in this article as NPC‐like is analogous to the terminology “chondroid metaplasia” or “mucous degeneration” as presented in other references (eg, Bergknut et al^97^ and Boos et al^94^).

^d^ Tears and clefts needs to be distinguished from processing artifacts. Nonartifactual tears and clefts commonly occur within the context of a degenerative milieu characterized by cellular and ECM metaplasia (eg, AF cells obtain an NPC morphology with increased AB staining of the surrounding ECM and/or presence of NPC clusters, cell loss or cell necrosis). Artifactual clefts tend to have sharp, jagged margins within regions of healthy cells and ECM. Scoring of tears and clefts may have to be considered within the context of method of lesion induction (ie, outer annulus surgical defect vs chemical nucleotomy).

##### Decalcification

In IVD tissues, the CEP and flanking bony portions of the vertebral bodies are ideally maintained for complete histological scoring of all IVD substructures. Decalcification prior to paraffin embedding is therefore essential for microtome sectioning. The decalcification method chosen can lead to a compromise between time requirements and preservation of tissue morphology, as the main difference between decalcification solutions is the speed of decalcification. Ethylenediaminetetraacetic acid (0.5 M EDTA) as a chelating agent is the method of choice for gentle decalcification, and generally the best at preserving tissue morphology^46^, ^89^ and is most compatible with immunohistochemistry. However, from weeks to months may be needed for EDTA decalcification, especially for large animal specimens. Alternative decal solutions and techniques are discussed in the Supporting Information (Appendix S1).

##### Mounting and staining

Thin paraffin‐embedded sections (5‐10 μm) should be mounted on positively charged glass slides (Supporting Information, Appendix S1). Due NP tissue's tendency to swell during the rehydration steps, which can cause wrinkles and reduce its adherence to the slide resulting in either poor quality sections (eg, folds) or even loss of sections, adherence to the slide can be enhanced by drying the sections overnight at 37°C prior to further processing, or precoating the slides with gelatin.^90^

**FIGURE 7:**
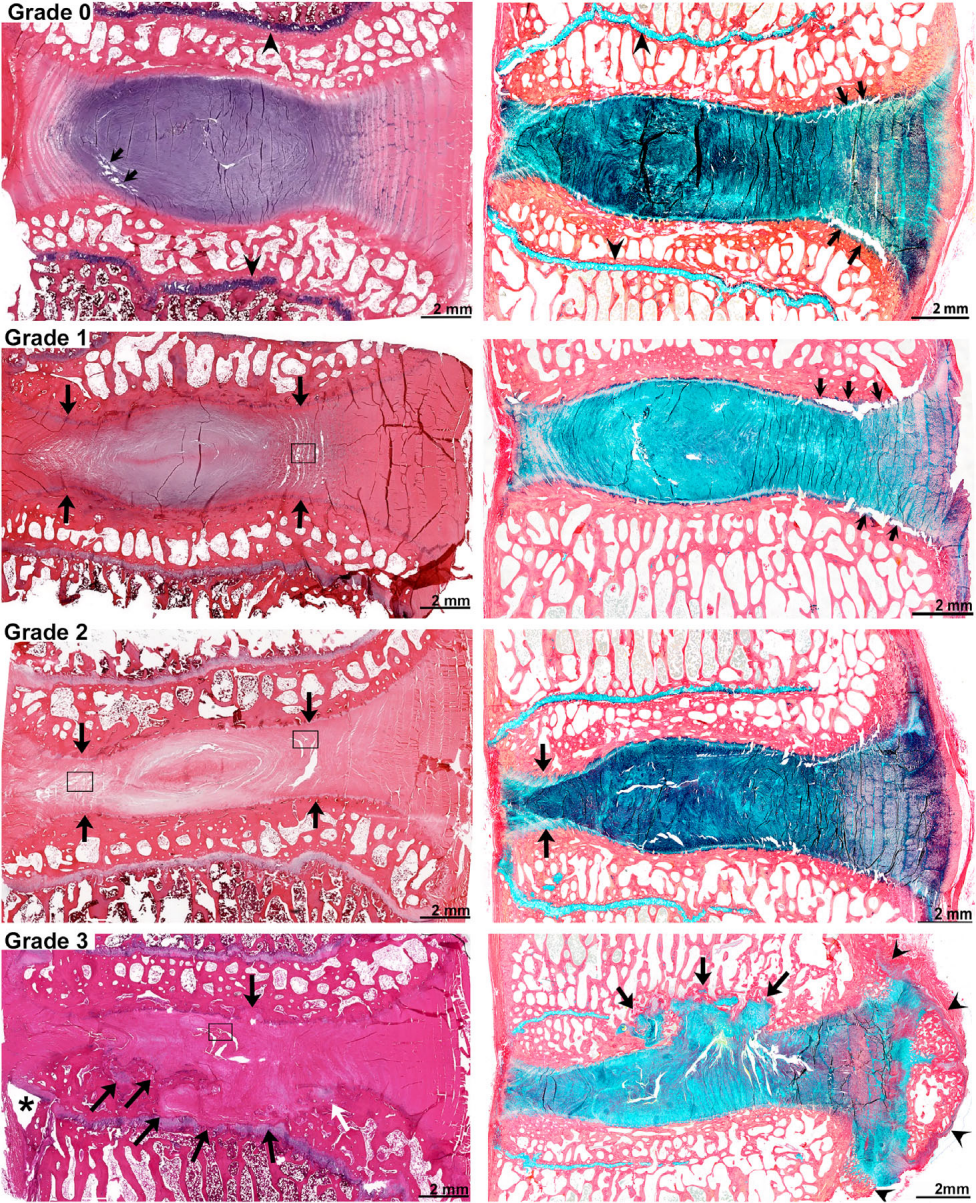
Composite depicting progressive stages of induced IVD degeneration in goat (left images, H&E) and sheep (right images bright‐field microscopy AB/PSR); mid‐sagittal lumbar spine sections, dorsal aspects of discs oriented to the left). IVD degeneration was enzymatically induced by chondroitinase‐ABC^†^ disc injection in 3‐year‐old, skeletally mature goats 12‐14 weeks prior to harvest. Nuclectomy was performed in 2‐4 year‐old, skeletally mature sheep 4‐5 months prior to harvest. ^†^Amsbio, Cambridge, MA. Grade 0 (normal, healthy controls) IVDs have a distinct nucleus pulposus (NP) that stains blue on H&E and deep turquoise on AB/PSR with a well‐defined NP‐annulus fibrosus (AF) interface. Hemi‐concentric well‐defined lamellae of the AF stain eosinophilic with H&E. The cartilage endplates (CEP) are thin uniform contiguous bands. The bony endplates (BEPs; stained pink on H&E and dark red on AB‐PSR) are comprised of uniform, regularly spaced arrays of bone trabeculae that are often flanked by persistent cartilage growth plates of the vertebral bodies (arrowheads). Section artifacts include clefts (short arrows) within the NP, AF or CEP interface that have sharp margins with abrupt transition to clear space devoid of degenerative matrix or cells (see Figure S2). Grade 1 (mild disc degeneration) IVDs show loss of basophilic/turquoise staining and reduced definition between the NP and AF that corresponds to reduced NP glycosaminoglycan (GAG) content and chondroid metaplasia of the AF, respectively. Inner to mid lamellae of the AF in this region (arrows) contain fine clefts spanned by proteoglycan‐rich fibrillated collagen corresponding to microtears (black frame, see Figure S2). CEPs retain their discrete, uniform contour but the trabecular bone of flanking BEPs has compacted (ie, endplate sclerosis). Grade 2 (moderate disc degeneration) changes show almost complete loss of NP basophilia (H&E) with poor definition of NP‐AF interface. Inner to mid lamellae of the AF in this region (arrows) show larger, more extensive clefts in the AF (black frames, see Figure S2) and progressive compaction of flanking trabecular bone. In the sheep, although proteoglycan staining of NP persists, there is loss of the inner to mid AF layers with NP protrusion into this region (arrows) that coincides to narrowing of the disc space and regional endplate thickening. Grade 3 (severe disc degeneration) changes include complete loss of NP basophilia (H&E) and more severely depleted NP GAG staining (AB‐PSR) with loss of NP architecture and poor discernment of NP‐AF interface. There is a collapse of IVD space and clefts within the distorted, degenerate NP (black frame, see Figure S2) and extrusion of degenerate chondroid IVD material beyond the CEPs that extends to the flanking cartilage growth plates of the vertebral bodies (arrows). The chondroid material (white arrow) stains dark blue on AB/PSR (see Figure S2). A triangular cleft at the dorsal aspect of the endplate (asterisk) is an artifact of sectioning. In the sheep, there is herniation of the ventral annulus with osteophytes that span cranial and caudal vertebral bodies (arrowheads). ^†^Amsbio, Cambridge, MA

**FIGURE 8:**
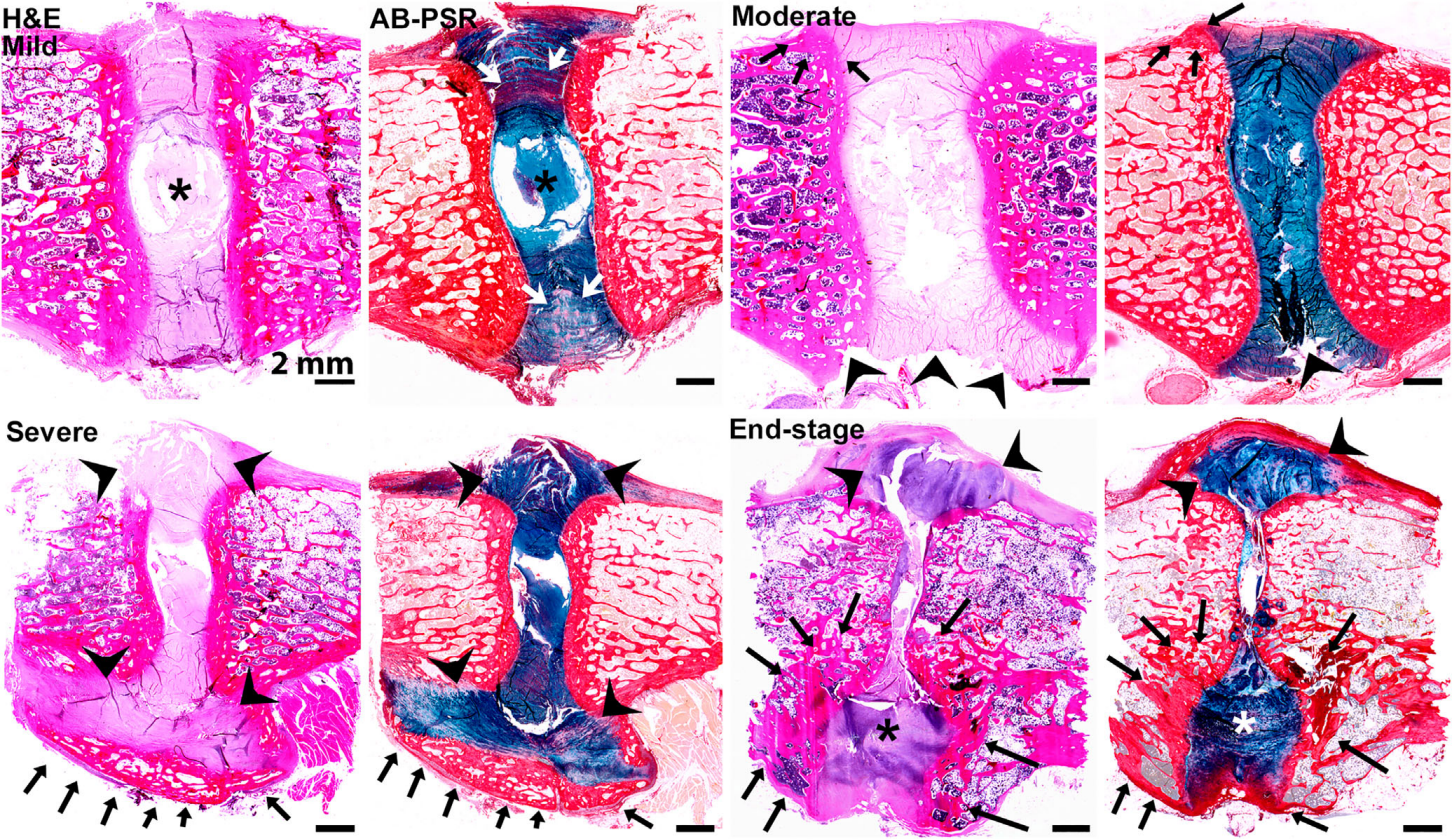
Low magnification composite of naturally occurring IVD degeneration. H&E and AB‐PSR stained bright‐field photomicrographs of mid‐sagittal sections of dog IVDs in various stages of naturally occurring degeneration. Mild shows degeneration and loss of glycosaminoglycan (GAG) staining within the nucleus pulposus (NP; asterisks) with increased GAG staining (chondroid metaplasia) of the annulus fibrosus (AF) and loss of definition. Moderate shows progressive NP and AF matrix degeneration with the production of small nodular exostoses (ie, syndesmophytes) at the dorsal margins of the AF (arrows); the ventral aspect of the H&E panel contains section artifact (arrowheads) and cannot be evaluated. Severe shows collapse of the IVD with partial dorsal and ventral extrusion of degenerate NP and AF matrix (arrowheads) and ventral bridging exostoses (arrows) compatible with intervertebral ankylosis (eg, self‐fusion). End‐stage IVDs show more complete extrusion of degenerate IVD matrix dorsally (arrowheads) and ventrally (asterisks) with complete collapse of IVD space and foci of bone‐to‐bone contact; ventrally, a large exostosis (arrows) surrounds the extruded IVD material (arrows). Reprinted with permission from Spine^152^ and further modified to serve the needs of demonstrating naturally occurring IVD degeneration changes. Note that these are representative images from a naturally occurring disc degeneration model and not from a large animal model where disc degeneration is induced either chemically or surgically. In the canine species the growth plates close and are as such absent in these sections indicating that they are from skeletally mature dogs

The standard histological staining for the general assessment of tissue structure and cell morphology is hematoxylin/eosin (H&E) that stains cell nuclei blue/purple (hematoxylin) and the collagenous ECM shades of pink (eosin) (Figures 7 and 8). Areas with high proteoglycan content (eg, NP) stain blue‐gray.^91^ Suggested histochemical stains for the evaluation of the IVD ECM are Alcian blue/picrosirius red (AB‐PSR)^92^ and safranin O/fast green.^91^ Alcian blue binds with negatively charged macromolecules in the ECM through electrostatic forces, thus stains sulfated and unsulfated GAGs blue at a pH of 2.5.^92^ Picrosirius red is an elongated birefringent molecule that complexes with collagen fibers and exploits the anisotropic properties of collagen fibers, staining fibers orange to red under standard bright field illumination, and enhancing their birefringence under polarized light, thus allowing for clear identification of collagen fiber structural organization or depletion.^91^, ^93^ Safranin O is a cationic dye that binds to the carboxyl and sulfate groups (negative charged) of GAGs.^91^ Fast green serves as a counterstain that stains GAG‐depleted areas green. Safranin O/fast green stain is commonly employed in cartilage pathology and as such could be employed to further study CEP/BEP histopathology. Toluidine blue, a cationic dye that stains GAGs with more intense staining due to its higher sulfur affinity than safranin O,^91^ counter‐stained with fast‐green is an alternative staining method. Toluidine blue‐fast green staining provides an additional advantage in that it can be evaluated by individuals with red‐green color vision deficiency, the most common type of color‐blindness. Because the alcian blue‐picrosirius red, safranin O or toluidine blue‐fast green staining intensity are relative and can vary between individuals of the same species, ideally staining should be conducted in a batched fashion, where all samples within a set are stained simultaneously under the same conditions (eg, concentration, timing, and temperature), and compared with a species‐matched internal control.^91^ For protocols and specific examples of these histochemical stains, see Supporting Information, Appendix S1.

**FIGURE 9:**
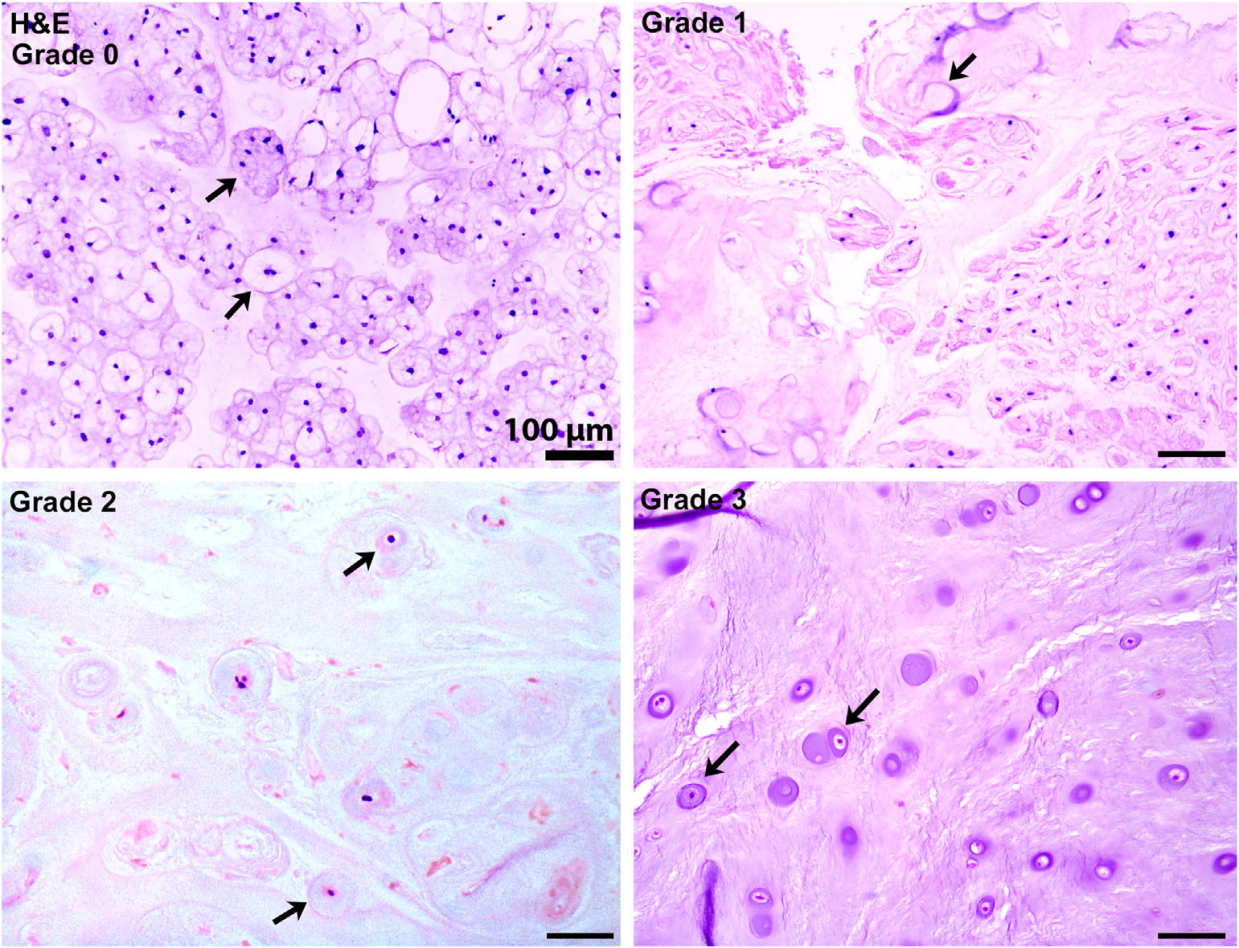
Nucleus pulposus notochordal cell depletion. H&E stained photomicrographs of progressive notochordal cell (NC) depletion within the nucleus pulposus (NP) in the dog IVD. Grade 0 shows that >90% of cells within the NP comprise a mixture of individualized and clusters of small and large vacuolated NCs (arrows). Grade 1 shows 30%‐40% NC depletion replaced by a population of nonvacuolated cells having large, eccentrically located, spindle‐shaped nuclei (arrow). Grade 2 shows degeneration and loss of nearly all vacuolated notochordal cells with remaining cells having centralized round hyperchromatic nuclei within lacunae (arrows). Grade 3 shows the complete loss of vacuolated NCs replaced by similar cells (arrows) as described for Grade 2

##### Histological grading scheme

Several histological grading schemes have been described in the literature for the different species (Supporting Information, Appendix S1), but to date there is no consensus on a histological grading scheme that is appropriate for all IVD large animal models. Therefore, to promote standardization and facilitate cross‐species comparisons of results, the authors have surveyed and reviewed the human and veterinary scientific literature to scrutinize all available schemes (including the human Boos^94^ and Rutges^95^) to synthesize a single comprehensive histological grading scheme that can be applied to commonly used large animal models (dog, pig, sheep, and goat; Table 4). The authors sought within the individually published schemes^40^, ^96^, ^97^, ^98^, ^99^, ^100^ comparable features of IVD degeneration shared across all species and incorporated scoring methods that provide uniformity as well as simplicity so that tissues could be evaluated by researchers without specialized pathology training, degrees, or board‐certifications.

The development of a semiquantitative four‐point grading scheme from 0 to 3 (Table 4; Figure 7, 8, 9, 10, 11, 12, 13, 14, 15, 16, 17, 18) separates scores into a scheme that corresponds to none, mild, moderate, and severe. Provided images are accompanied by different histological subscores to demonstrate histologic changes that are not necessarily dependent on or restricted to any given species, breed, age, method of induction, or time frame. Since there is not yet a consensus on which parameter might contribute more or less to the overall degenerative process or clinical outcome, in this newly developed scheme, all categories have a score of 0‐3 so that all parameters contribute equally to the final score. The authors recognize that histologic scoring system without the use of quantitative image analysis is inherently subjective, and therefore grading schemes that showed good consistency and repeatability for intra‐ and interobserver scores^97^ were included. The authors emphasize that this uniform grading scheme has benefits in evaluating components of the IVD (NP, AF, and CEP) as separate entities with respect to their architecture and cellular/ECM components, and can be used to evaluate changes associated with different experimental models (ie, lesion induction method). The authors recognize that grading schemes may not apply, in part or in total, to certain experimental models or research questions and that modifications of published grading schemes are common and often appropriate. New insights on IVD histopathology and comparative analysis across species, including human, would need to be considered for further improvement of the scoring scheme in the future. Consultation with a highly experienced IVD expert or veterinary pathologist can provide important insights and assistance in modifying or developing an appropriate scheme to best fit the model. For example, in models that develop significant inflammation, given the limited inflammatory reaction typically present within IVD degeneration where ECM extrusion is not present, inflammatory infiltrates may indicate either postsurgical infection or a type of hypersensitivity reaction specific to the surgical modifications or implant devices/materials.^101^ In this case, the authors suggest that researchers develop an additional component to the grading scheme that encompasses the spectra of inflammatory changes specific^102^ to the model. Moreover, note that new bone formation and subchondral bone sclerosis are best determined and/or quantified via imaging (plain radiography and μCT), since for histological analysis only one specific location is analyzed, and therefore the severity or extent of vertebral osteophytosis and sclerosis can be misinterpreted. Other aspects of IVD pathology not included in this uniform scheme include, in addition to inflammation, neovascularization, and nerve invasion/fiber characterization. These parameters will need to be evaluated on a case‐by‐case basis and will depend on the research question posed.

##### Immunostaining

Immunohistochemistry (IHC) and immunofluorescence are applied to identify specific cellular or extracellular proteins in the tissue. Immunostaining is particularly challenging for large animal studies, as most commercially available antibodies are developed to react with antigens from human and rodent proteins. Cross‐reactivity with the particular species, including appropriate positive and negative controls, needs to be confirmed to ensure specific immunostaining. Furthermore, decalcification and antigen retrieval present additional challenges for obtaining high‐quality and consistent immunostaining. Within this context, immunostaining procedures need to be optimized from the first step to achieve satisfactory and reproducible results. The spine researcher undertaking immunostaining is referred to detailed information provided in the (Supporting Information, Appendix S1) and to Binch et al reporting on these protocols for human samples.^103^

### 1.8 Biochemical, gene, and protein analysis

For this analysis, the NP and AF tissue (from ¼ IVD; Figure 2B) is carefully separated, cut into small pieces, snap frozen in dry ice or liquid nitrogen, and stored at −80°C until required for biochemical analysis and/or biomolecular analysis. For gene expression analysis, RNAlater solution is suitable to protect cellular RNA if the tissue cannot immediately be processed or snap frozen. If other bioactive components need to be measured from tissue extracts, the tissues can be collected in a purpose‐specialized lysis buffer that extracts and protects the proteins to be measured, homogenized, and stored at −80°C until biochemical analysis to extract growth factors/cytokines for measurements. Prior to tissue digestion, samples are weighed and then dried (eg, lyophilization or oven) in order to normalize biochemical data for wet weight. Thereafter, the supernatant and pellet can be enzymatically digested overnight, and the DNA and GAG content can be measured.^18^, ^104^ A hydroxyproline assay can be used to determine the digested samples hydroxyproline/collagen content.^105^ Note that these are merely quantitative assays that do not provide information on the ECM quality in terms of GAG or collagen integrity. As alternatives to wet or dry weight for normalization, results may also be normalized to total protein, cell number, or DNA content.

For gene expression profiling, the specific challenges of cartilaginous tissues like the NP include low cellularity, difficult homogenization, and ECM rich in glycosaminoglycans that are copurified during extraction and interfere with the RT‐qPCR reaction.^106^ Tissue homogenization can be achieved with variable methods utilizing either mincing, cryo‐sectioning, or cryo‐pulverization. RNA extraction techniques can be optimized to ensure satisfactory RNA yield and quality from GAG‐rich tissues, for example, via additional enzymatic digestion steps upon collection of the tissue or high salt coprecipitation of GAGs during the RNA extraction process.^107^, ^108^, ^109^ In the case of limited starting material, unbiased preamplification of the target sequences may provide an alternative.^18^, ^110^ Although the authors anticipate that these techniques may also be suitable for other species, age groups and IVD levels than those employed in the reported work, the RNA isolation method would need to be validated to make final conclusions. To normalize target gene expressions, it is suggested to use more than one stably expressed reference gene. Gene expression profiling is a tool that is often used to address the findings from a mechanistic perspective and correct normalization of these profiles is essential. To date, while sets of stable reference genes have been studied for some human, dog and goat IVD tissues,^18^, ^106^, ^111^ they remain to be determined for most species and tissues. As gene expression levels are not always correlated with protein levels, parallel quantification of cytokine, chemokines, ECM molecules, and other proteins of interest in the tissue extracts employing Western blots and/or commercially available testing kits, ELISAs or multiplex immunoassays is, in the authors opinion, advantageous.

### 1.9 Clinical scale

At present, there are no methods established solely for the purpose of assessing or quantifying spinal pain in large animal models. However, behavioral and physiologic parameters can be used to evaluate the degree of pain and responses to analgesia or therapies.^112^ Behavioral or physiologic parameters that can produce quantifiable outcome measurements can be included a priori during study design. In this article, we provide an overview of behaviors and clinical parameters that may be included to evaluate research outcomes and instruct future recommendations for relevant preclinical outcome measures in large animal models.

#### 1.9.1 General pain assessment of large animal models

To date, there is a relative paucity of data regarding assessment of pain in preclinical large animal models (Figure 1) and even in animal models in general^8^ despite the fact that pain is the most clinically relevant parameter for patients suffering from spinal conditions. While small animal pain models are available^113^ based on a single IVD level intervention, they remain largely qualitative for preclinical large animal models and have not yet been properly validated specifically for back pain related to IVD degeneration.

Recognizing pain and assessing its intensity and duration are essential to ethical hypothesis‐driven research as clinically relevant outcome measures, as a potential source of experimental error, and as a key component of animal welfare.^114^ In addition to recognition of pain, pain management with effective analgesia and humane endpoints for uncontrollable pain during an experimental study are required components of the *3R principle* (*Replacement*, *Reduction*, and *Refinement*). Pain is the result of complex combinations of physiological, immunological, cognitive, and behavioral parameters. As such, pain may be generated by causes other than known interventions, including husbandry and aging related factors that could skew experimental data. Therefore, behavioral indices (Table 5) and careful extrapolation from the clinical presentations should be considered when assessing pain in animals.^114^, ^115^

**TABLE 5:** General assessments and behavioral/physiological indicators of pain that apply to large animal models

*General considerations when conducting assessments of pain in large animal models*
The safety of laboratory personnel and animals should always be maintained as a high priority. The same laboratory personnel should conduct assessments of the animals for the duration of the study. Specific training needs to be provided so that scoring can be consistent. Baseline assessments of pain should be determined after adequate acclimation to the experimental housing, environment, and conditions.^195^ Observe the animal's behavior without disturbing it first (see behavioral indices). Consider that the animal's behavior can change drastically in the presence of a new observer or characteristics (ie, observer's sex, perfume, glasses, hat, color of clothes, etc.). Examine the entire animal prior to focusing on areas of anticipated or perceived pain. Initially assess for pain by gently palpating or handling presumed painful areas before performing any approved provocative maneuvers. Monitor accurate weights and food consumption for each animal. Also, note any changes in micturition or defecation. Determine and communicate definitive criteria regarding definitions, measures, and levels of pain requiring intervention along with specific actions in response prior to study initiation.
*Behavioral/Physiological indices*
*General behaviors* Agitation, restlessness Recumbency and immobility Isolation, social desynchronization Aggressiveness Loss of appetite (weight loss, decreased urinary/fecal output)
*Physiological changes* Heart rate Respiratory rate Blood pressure Internal temperature
*Calls/Vocalization* Number and duration Intensity Spectral composition
*Postures/gait* Posture changes (e.g., hunched) Tonic immobility, stiffness Locomotion/Lameness/Gait changes
*Altered behavior/mutilation* Trembling, Spasm Scratching, licking, biting with localized pain Withdrawal reflex Shaking

Behavioral indicators of pain are often markedly different for laboratory animals when compared to domesticated animals of the same species. As such species‐specific pain indicators are required to successfully incorporate behavioral and/or nociceptive indicators as measurable data. Perception of pain, nociceptive response generators, and clinical presentations of pain can be species dependent. There are no generally accepted methods or objective criteria for assessing pain in large animal models employed for IVD regeneration. However, a detailed neurologic examination by a veterinary expert can help localize spinal pain.^1^ Due to the highly specific nature of research investigations, the optimal pain scale for a given experiment should ideally be as objective, standardized, and repeatable as is possible based on species, methods, and resources as determined *a priori* using generally accepted or validated methods.

#### 1.9.2 Quantitative measures of pain

While limited, there are analyses that can produce objective quantitative data assessing pain and/or functional deficits in large animal models. One suggested measure is the Von Frey test, which has been used widely and validated in several species including dog, pig, sheep, young goats, and humans^116^ to assess the mechanical nociceptive threshold.^117^, ^118^, ^119^ Von Frey filaments are applied to the skin to deliver nociceptive mechanical stimulation with incrementing levels of force, where upon the withdrawal threshold is determined. Depending on severity, duration, and degree of neurologic deficit, Von Frey thresholds may increase or decrease.

Gait analysis can be another quantitative tool for lameness^120^ caused by IVD‐related back pain. There are several different methods to perform gait analysis including kinematic gait analysis, kinetic gait analysis (force plate), and temporospatial gait analysis (pressure sensing walkways). Pain can be highly variable among species, strain/breeds, and even individuals in the same cohort.^121^, ^122^ It can also vary greatly based on study‐specific interventions. As such, animals are acclimatized to the environment and the measurement method and trained for consistent gait analysis; baseline measurements based on at least three‐day recordings prior to the intervention can correct for individual variances. Objective gait analysis specifically for low back pain has not been applied in large animal models and is in its infancy even in the veterinary clinical field.

Lastly, general activity can be monitored using commercially available activity monitoring devices,^123^ external/implantable telemetry,^124^ and/or video analysis.^125^ These methods provide ways to quantify general activity level which is expected to decrease with increased pain perception in animals. Especially, external/implantable telemetry has been used widely in large animals in various research settings as some telemetry devices have capabilities to record and store detailed and continuous physiologic parameters such as heart rate, ECG, respiration, and glucose levels along with activity level. There are advantages and disadvantages of each activity monitoring method that pertain to the purpose of study.

#### 1.9.3 Species‐specific recognition and assessment of pain

Assessment of pain in laboratory pigs can be challenging as mature pigs do not tend to show markedly altered behavioral indicators. Pain assessment is better established for piglets as they are employed extensively in the production and research settings. The Pig Grimace Scale (PGS) has been developed for use in piglets.^126^ To date, there are no published behavior indices specifically for orthopedic experimental models in swine. Therefore, pain assessment categories need to be developed based on individual projects with considerations of the Pig Grimace Scale and other general physiological and behavior cues^127^ for pain in pigs.

Even though their behavior is different, general signs of pain in laboratory sheep and goats are similar. Sheep have been reported to tolerate severe injury without overt signs of pain or distress.^114^ Pain can lead to the cessation of rumination, eating, and drinking. Curling of the lips has been observed as a reliable indicator of pain. Goats are more likely to vocalize with pain and may grind their teeth. Increased agitation (foot stomping) and frequent changes in posture could indicate pain. Especially in dairy breeds of goat and sheep, rapid decrease in milk production can be observed along with body weight loss and decreased body condition. The Sheep Grimace Scale (SGS) has been established for use in laboratory sheep following orthopedic procedures (unilateral tibia osteotomy)^128^ and pain generated by hoof abnormalities.^129^

#### 1.9.4 Recognition and assessment of pain in dogs and clinical grading

Dogs in pain^115^ often appear stiff or are unwilling to move when aroused. They may stop greeting research or husbandry staff members and may consistently retreat to the extents or protected areas of their housing. Dogs can also exhibit pain by being restless, agitated, and hypersensitive. Some dogs may attempt to bury or hide food when painful. Dogs in pain may bite when painful areas are palpated, which can be mistaken for aggressive behavior. Vocalization can be evident; however, excessive barking is unlikely in the presence of pain. Growling, whimpering, or howling are indicators of pain in dogs; however, neither lack of vocalization nor excessive vocalization is a reliable indicator of pain. There is no published or validated grimace scale for laboratory dogs to the authors' knowledge. However, there are several validated pain assessment methods for use in client‐owned dogs including The Melbourne Pain Scale, MPS; Colorado State University Canine Acute Pain Scale; The Canine Brief Pain Inventory, CBPI; Helsinki Chronic Pain Index, HCPI; Canine Orthopedic Index, COI; Glasgow Composite Pain Scale, GCPS),^130^, ^131^, ^132^, ^133^ which may have utility for application to laboratory dogs when the differences are considered.

Within the context of large animal models, spontaneously occurring disorders of the spine in client‐owned dogs are common and can serve as effective models for translational research with simultaneous benefit to pets and pet owners. Both chondrodystrophic (CD) and nonchondrodystrophic (NCD) breeds spontaneously develop IVD disease. The onset and anatomic location of IVD disease are different and this provides opportunities to model different IVD disease phenotypes. A brief overview of the most common clinical entities in canine veterinary patients is outlined in Table 6. Researchers are encouraged to discuss with veterinary specialists in identifying the most appropriate clinical entity to be employed in their translational endeavor once the intended approach has been proven to be safe and have sufficient efficacy. In addition to the pain assessment methods, in clinical patients, gait/posture analysis can be performed to monitor changes due to IVD perturbations. Pain associated with cervical and thoracolumbar IVD disease (spontaneous or induced) can be assessed via neurologic grading of IVD disease. Table 7 shows the most commonly used clinical scales when assessing IVD disease in dogs. As the clinical grade increases, clinical signs become worse with decreasing normal functions. Also, higher grades indicate severity of impacts on PNS and/or CNS and worse prognosis for return to normal functions. This particular scoring scheme can be extrapolated to the other large animal models.

**TABLE 6:** Spontaneous IVD diseases and common clinical signs in client‐owned dogs

CD	NCD
Cervical‐Acute herniation—C2‐C5 Clinical signs may include hyperesthesia of the neck and forelimbs, muscle spasms and pain in the cervical musculature, paresis/paralysis of all limbs^196^	Cervical‐chronic protrusion—C5‐C7; Wobbler Syndrome caused by cervical vertebral instability Clinical signs are related to the dynamic movement and compression of spinalcord and nerve roots by protruded cervical IVD. Nerve root signature (pain apparent on palpation or traction of the limb)
Thoracolumbar‐Acute herniation—T11‐L3 Acute pain that may be accompanied by motor deficits in the hindquarters depending on the level of spinal cord trauma. Damage to the upper motor neuron causes the urinary bladder to be full and tense which leads to difficulty with expression of the urinary bladder eventually leading to urinary incontinence.	Cauda Equina Syndrome—L6‐L7‐S1, chronic protrusion^197^ Chronic pain with acute episodes, progressive hind limb weakness and muscle wasting. May lead to progressive sensory and motor deficits in the hind limbs. Nerve root signature (pain apparent on palpation or traction of the limb). Damage to the lower motor neurons will influence bladder function that will cause urinary bladder to be flaccid, eventually leading to urinary incontinence and fecal incontinence.

*Notes*: Dog breeds can be categorized into chondrodystrophic (CD) or non‐chondrodystrophic (NCD) breeds. CD breeds have distinctive conformational differences that are characterized by short limbs and long torso length compared to limb strengths. The clinical signs relate to the fact that the diseased IVD is affecting the peripheral nervous system (PNS) or central nervous system (CNS). Localization is done based on thorough clinical and neurological examination complemented by imaging modalities.

**TABLE 7:** Neurologic Grading of IVD disease

Clinical grade	Representation
0	Normal gait
1	Normal gait, cervical or thoracolumbar pain, hyperesthesia
2	Ambulatory paraparesis with decreased proprioception
3	Non‐ambulatory paraparesis (unable to walk or stand unassisted) with absent proprioception
4	Paralysis (not able to stand or walk), +/− Urinary incontinence, conscious deep pain perception present
5	Paralysis, conscious deep pain perception absent, urinary, and fecal incontinence

*Notes*: As the clinical grade increases, clinical signs become more severe and prognosis for regaining affected functions worsens. While this scheme has been specifically reported for scoring of the neurologic grade in canine species that suffer from clinical disc‐disease, the representation can be extrapolated to other large animal models involving the species‐specific pain indicators.

### 1.10 Perspective—future outlook

The current manuscript presents a comprehensive tool box for conducting *in vivo* large animal studies of IVD treatment with the focus on methodology and complementary outcome parameters (key points summarized in Table 8). Moving forward, key areas for refinement and expansion with specific focus on the comprehensive tool box are discussed below to facilitate comprehensive analysis and a better understanding of the underlying pathology and model‐specific differences.

**TABLE 8:** Highlights of the comprehensive tool box for large animal studies in IVD degeneration and regeneration

*Macroscopic scale*
Gross morphological grading is relatively low cost, easy to implement, and provides overall information on IVD macrostructure. Imaging via radiography is a rapid and inexpensive modality Radiography can be complemented by MRI to obtain the Disc Height Index and detailed information on degenerative changes in vivo. T2‐weighed images are species‐dependent as notochordal cell‐rich IVDs have a higher signal intensity. Non‐destructive biomechanical tests allow functional evaluation of the IVD.
*Microscopic scale*
A new four‐point histological grading scheme with consensus for all large animal IVD models has been compiled, based on validated schemes for individual species. On mid‐sagittal sections all relevant IVD components: the nucleus pulposus, the annulus fibrosus, and the cartilage endplates can be evaluated H&E for cell morphology and extracellular matrix strains enable microscopic IVD grading.
*Clinical scale*
Following the ARRIVE guidelines and consultancy with veterinary and animal welfare experts helps determine pain‐related outcome measures, analgesia rescue protocols, and humane endpoints. Client‐owned dog patients represent different clinical entities that could contribute to translational research and lead to the development of therapeutic strategies. Post‐operative evaluations for neurologic state, pain, and other clinical parameters may be species‐specific. It is beneficial to have a standard assessment scheme that will decrease inter‐evaluator variability.

The most significant species‐specific confounders affecting outcomes and their utilization include sex and breed‐dependent genetic background. IVD (patho)physiology is potentially influenced by sex hormone differences and as well as related to the species‐dependent reproduction cycle, particularly considering the well described effects of sex on bone (patho)physiology within the field of osteoporosis.^134^ As such, where possible involvement of both sexes in studies will allow discernment of this effect. In small animal models, strains of different genetic backgrounds are beginning to be employed for a better understanding of IVD aging and pathology^135^, ^136^ and genetic background has been shown to play a role in rodent models^137^; however, these remain relatively unexplored in large animal models. There is the well described role of the FGFR4 retrogene in the canine species,^138^ and livestock animal models typically involve genetic polymorphisms or mutations affecting skeletal developments. The IVD may potentially show similar genetic traits, but only their effects on bone phenotype have been described so far. An example is the quantitative trait locus mapping to the growth differentiation factor‐8 (GDF8 or myostatin) gene reported in Texel sheep^139^, ^140^ and in cross‐breeds like the Swifters, with GDF8 shown to inhibit chondrogenesis by suppressing SOX9 and collagen type II expression.^141^ Within this context, there are reports of spontaneous NP^18^, ^142^, ^143^, ^144^, ^145^, ^146^ and AF^147^, ^148^ repair in large animal models. While those may partly relate to IVD size and as such to nutritional status of the IVD,^149^ to date there is limited understanding of the differences in intrinsic repair capacity across large animal species and how these translate into changes in the outcomes of the three scales, that is, macroscopic, microscopic, and clinical, displayed in this manuscript.

With the exception of dogs, IVD geometries have been reported for goats, pigs and sheep^5^, ^6^, ^7^, ^150^ and summarized by Fusellier et al.^151^ Furthermore, varying measurement methodologies (eg, application of axial load) makes comparison between the available studies difficult. Although basic geometries have been documented, anatomical details are still insufficient. For example, CEP‐thickness for dog,^152^ pig,^153^ and human^154^ were reported, but not for goat and sheep, and these may be important when selecting models for investigation of IVD nutrient transport. Furthermore, potential damage to the CEP (eg, due to freezing or enzymes^155^, ^156^) and its role in degeneration needs to be further investigated. For future studies, one might also consider freezing specimens comparable to cryopreservation for total IVD transplantation.^51^

Macroscopic grading based on gross morphology and MRI imaging are simplified and accepted as five‐point scale systems enabling overall scoring and subsequent reporting of IVD degeneration across large animal models and humans. However, because of their coarse nature, to preserve their efficacy and enable cross‐study comparisons, liberal adaptations of these schemes are preferably avoided. Furthermore, macroscopic grading is ideally complemented by more detailed scoring and analysis of the different degenerative processes at the microscopic or biochemical level. The degenerative processes of the different components of the IVD (NP, AF, cartilage, and bony EPs) do not necessarily go together for all the studied parameters and may also progress differently depending on the model used to induce IVD degeneration.

In this respect, CEP, BEP, and the vertebral body changes are to date relatively understudied while they have been related to IVD pathobiology,^157^, ^158^ and prolonged low back pain and disability.^43^ Macroscopic and microscopic outcome parameters can contribute to improving the comprehension of BEP/CEP and vertebral body pathology and need to be further improved and expanded based on the new scoring scheme of human IVD degeneration.^159^ Within the basic set of parameters, macroscopic grading of mid‐sagittal planes, even though more cumbersome in its processing due to challenges in the decalcification processes and subsequent paraffin sectioning can quantify changes of the CEP and vertebral bone. Furthermore, MRI imaging produces indispensable data by allowing longitudinal investigation of experimental manipulations in large animal models. It enables evaluation of the diffusion capacity of the EP and bone marrow lesions of the vertebral bodies associated with degenerated IVDs, known as “Modic changes” described in human literature. Modic changes have been commonly associated with neck^160^ and low back pain,^161^ and with IVD degeneration^43^, ^162^, ^163^, ^164^, ^165^; however, identification and classification can depend on the MRI field strength,^157^ and to date they remain relatively unexplored in large animal models.^18^, ^166^, ^167^ These advanced imaging parameters could be further explored as they may shed more information on the therapeutic potency of the employed experimental strategies in regressing Modic changes.

In addition to Modic changes, MR imaging, especially advanced techniques, assists in improving the in‐depth knowledge of IVD degeneration while facilitating the 3Rs principles. Furthermore, considering that the degenerative changes scored on MRI are a continuum rather than the predefined five grades, the subjectivity of a five‐grade scoring system could be further improved with the aid of artificial intelligence and machine learning currently available for human applications.^168^, ^169^ Sensitivity of standard signal intensity‐based MRI is limited in early grades of degeneration and may lead to a gap in the recognition of early tissue alterations occurring in large animal models. To this end, several biochemically sensitive MRI techniques have been developed to overcome these limitations in human IVD degeneration and have thus far only been limited used in large animal studies. Those include advanced MR imaging intended to evaluate and establish relations of IVD degeneration with water content, the collagenous ECM structure, or proteoglycan content, including T2 mapping,^170^T1rho‐weighted,^171^ and sodium imaging,^172^ delayed gadolinium‐enhanced magnetic resonance imaging (dGEMRIC)^173^ and the ultrashort time‐to‐echo (UTE) MRI technique^174^ to assess the cartilaginous endplate, and chemical exchange saturation transfer imaging (CEST)‐based imaging to determine GAGs^41^ and pH^175^ changes within the IVD tissue. Although these techniques still have the limitation of requiring complex equipment and training, it is anticipated that they will play an increasingly important role in defining imaging biomarkers that relate to lower back pain and thereby complementing the outcomes of future large animal studies.

Bony changes can be studied in a qualitative and quantitative manner with plain radiography, and postmortem via μCT or high‐resolution peripheral quantitative computed tomography (HR‐pQCT) thereby providing a more complete representation of bone pathology of the entire IVD segment studied (eg, focal vs diffuse endplate sclerosis, extent of osteophytosis, or structural abnormalities in the adjacent vertebral bodies associated with altered remodeling), as compared to histology, which is limited to one thin section. Within this context, in the histopathological grading scheme, scoring for bony endplate sclerosis is no longer integrated and scoring for new bone formation (eg, exostosis or osteophytosis) without radiologic correlation may provide an inaccurate representation, especially if no changes are detected in the examined section(s). While guidelines for assessing the bone microstructure have been defined for rodents,^176^ these need to be fine‐tuned for the purpose of assessing bone microstructure and the BEP in large animal models. Correlation of μCT with other parameters like histological measurements of the BEP has been reported in the pig^177^ and goat,^100^ showing this imaging technique can provide valuable information regarding the IVD degeneration process. In the future, morphometrical measurements provided by μCT could be used to define and evaluate the overall architecture of the spine segment including IVD height,^179^ vertebral bone mineral density and architecture, and changes of the BEP and vertebrae.^100^, ^178^

The presented histological grading scheme with consensus for all four considered large animal IVD model species was based on individual, validated schemes and helps to compare inter‐study results regardless of the experimental model employed (ie, large animal species or method of lesion induction). This newly developed scheme can be used by researchers without specialized pathology training. Moreover, all categories have a score of 0‐3 (healthy, mild, moderate, severe degeneration) so that all parameters contribute equally to the final score. The authors propose this scheme as a starting point based on the available literature on large animal models, although new insights in IVD histopathology and comparative analysis across species, including man, should be considered for further improvement of the scoring scheme. In the authors' opinion, until more information becomes available or a consensus evolves that correlate specific histologic parameters with clinical disease or outcomes, that the newly developed scheme improves objectivity compared with previous histologic grading schemes which contained unequal categories,^40^, ^96^, ^97^ enabling certain categories to outweigh the others. When studying regeneration in large models of disc degeneration, it remains to be determined which changes, currently interpreted as degenerative reactive changes, may be species‐dependent relating to a differential intrinsic repair capacity. Within this context, specific histological features, like NP cell clustering, could be the result of a regenerative response.^180^For histological and immunohistochemical evaluation, the possibility of automation is increasingly implemented to provide more objective assessment compared to the conventional grading that may depend on the individual observer. Nevertheless, automated methods need to be precisely described and validated, as they are susceptible to artifacts. With emerging artificial intelligence and machine learning technologies, misinterpretations by automated evaluations will be further diminished.

Employing consistent methodology is imperative for proper comparative meta‐analysis of the generated data to evaluate translatability of therapeutic. However, the relationship between structural tissue changes, function of the IVD, and clinical disease remains a topic of debate. With respect to characterizing structural changes of degenerated and repaired IVD tissues, GAG and collagen composition are reported based on the basic set of measurements (ie, stains and quantification by the DMMB and HYP assays). However, these methods do not give insight into quality of the ECM that is reflected in the type of these macromolecules and lengths of the proteoglycan and glycosaminoglycan chains present. Therefore, mass spectrometry, high‐performance liquid chromatography, gel chromatography, sodium dodecyl sulfate‐polyacrylamide gel electrophoresis (SDS‐PAGE), agarose gels, and Western blots, for example, help with thorough characterization.^181^ Alterations in the polydispersity of aggrecan^182^ and fragmentation of small leucine‐rich proteoglycans, biglycan, and fibromodulin^183^, ^184^ have been reported with degeneration. Likewise, hydroxyproline assays can potentially be coupled with qualitative analysis (eg, immunostaining) to determine total collagen content and distinguish among the different types of collagen present. Furthermore, similar analytical methods apply to de novo engineered tissue to better understand the full spectrum of the regenerative strategy on proteoglycans, collagens, and other noncollagenous proteins. Finally, even though the nucleus‐endplate integration as well as the interlamellar cohesion and elastic network were described,^185^, ^186^, ^187^ they are seldom studied in degeneration and regeneration studies.

While it is generally accepted that in progressive IVD degeneration structural changes compromise IVD biomechanical properties resulting in the long‐term in pain, biomechanical tests are rarely performed when investigating regenerative strategies. This is likely due to limited resources or technical support. With the proposed algorithm, nondestructive mechanical evaluation is feasible—with complexity depending on the available equipment. However, comparative studies of large animals are still needed to deepen the existing knowledge and increase translatability. For example, IVDs with notochordal cell‐rich NPs (pigs, NCD dogs) differ significantly regarding spine biomechanical properties, which are proposed to be in relation with the different cell types present in the NP and inherent differences in ECM composition.^188^ Nonetheless, it has also been suggested that their AFs do not differ substantially from the other large animal species.^10^ However, it has yet to be determined exactly what contributes to differences in clinical presentation and lesion progression between CD and NCD dogs. Further exploration of the correlation between ECM composition, IVD biomechanics, and lesion morphology between these two spontaneous dog models could help elucidate the biomechanical effects of IVD lesions and ECM composition in humans.

Changes at the macroscopic level have been reported to be more prevalent in patients suffering from back pain^189^ but they are also present in asymptomatic patients.^190^ As such, in the authors opinion, study designs addressing only the macroscopic and microscopic parameters of IVD degeneration/regeneration do not necessarily determine the clinical relevance of the therapeutic strategy being studied. Herein, behavioral and/or pain outcome measures are reliable indicators of how effective candidate therapeutic interventions may be. There are better tools becoming available with the advancement of technology such as motion analysis and telemetry, which will permit access to highly detailed and quantifiable data without interfering with the wellbeing of the laboratory animals. Furthermore, collaboration with veterinary experts to establish clinical parameters relevant to the spinal pathology being studied, for example, acute thoracolumbar IVD herniation in the CD dog or progressive lumbosacral IVD protrusion in the large breed working dog, that are relevant to the individual studies is valuable.

In conclusion, this comprehensive tool box for large animal IVD models comprised of macroscopic, microscopic and clinical outcome parameters can assist in generating a comprehensive data set to address the full spectrum of changes associated with IVD degeneration and regeneration in studies with large animal models. Within this context, there are opportunities for further refinement and expansion, including the implementation of the emerging innovative techniques we have touched on throughout this article. By employing such an algorithm, metadata analysis will be less complicated, even across species, and provide insight into model‐dependent differences and how they could translate to understanding human pathology, improving translatability and clinical relevance which will ultimately serve patients suffering from pain and disability due to IVD degeneration.

## CONFLICT OF INTEREST

The authors have the following declarations: Naomi N. Lee; Elias Salzer; Andres F. Bonilla; Frances C. Bach; Julie B. Engiles; Andrea Vernengo: No conflicts to declare. James L. Cook: Artelon: Paid consultant Arthrex, Inc: IP royalties; Paid consultant; Paid presenter or speaker; Research support AthleteIQ: IP royalties ConforMIS: Research support CONMED Linvatec: Paid consultant Coulter Foundation: Research support DePuy Synthes, A Johnson & Johnson Company: Research support Eli Lilly: Paid consultant; Research support *Journal of Knee Surgery*: Editorial or governing board Merial: Research support Midwest Transplant Network: Board or committee member Musculoskeletal Transplant Foundation: Board or committee member; IP royalties; Research support National Institutes of Health (NIAMS & NICHD): Research support Purina: Research support Schwartz Biomedical: Paid consultant Thieme: Publishing royalties, financial or material support Trupanion: Paid consultant U.S. Department of Defense: Research support Zimmer‐Biomet: Research support. Sibylle Grad; Zulma Gazit: JOR Spine—scientific advisory board. Keita Ito: NC Biomatrix: Paid consultant and shareholder; *Global Spine Journal*: Deputy editor: Biomechanics and Modeling in Mechanobiology: Editorial board. Lachlan J. Smith: JOR Spine—scientific advisory board; PLOS One—academic editorial board member; Connective Tissue Research—Associate Editor; National MPS Society—Scientific Advisory Board; ORS Spine Section—committee member. Hans‐Joachim Wilke: Grammer AG: Paid consultant; *European Spine Journal*: Deputy Editor. German Spine Foundation: Vice President. Marianna A. Tryfonidou: *JOR Spine*—scientific advisory board; SentryX—scientific advisor.

## AUTHORS' CONTRIBUTIONS

All authors: conceived set up, drafted in working groups parts of the manuscript (macro scale: Elias Salzer, Andres F. Bonilla, Zulma Gazit, Keita Ito, Lachlan J. Smith, Hans‐Joachim Wilke; micro scale: Frances C. Bach, Sibylle Grad, Andrea Vernengo, Julie B. Engiles, Marianna A. Tryfonidou; clinical scale: Naomi N. Lee, James L. Cook, Marianna A. Tryfonidou and provided supportive material for the different sections. All authors revised the whole manuscript critically. Naomi N. Lee, Elias Salzer, Julie B. Engiles, Marianna A. Tryfonidou: coordinated the drafting efforts; did substantial contributions to guideline design, acquisition, analysis, and interpretation of current literature. All authors have read and approved the final submitted manuscript.

## Supporting information


**Appendix** **S1:** Supporting Information

## Endnote

^1^
For examples see http://www.cvmbs.colostate.edu/vetneuro/Neuro_exam/neuroExam.html and https://www.vetneuro.com/images//forms/Neuro-Exam-Form-1-24-19.pdf
