# Supplementary material for: A comprehensive tool box for large animal studies of intervertebral disc degeneration
Source: JOR Spine. 2021 Jun 14;4(2):e1162. doi: 10.1002/jsp2.1162 (PMC8313180; doi:10.1002/jsp2.1162)
Supplement: Supplementary file 1 — Appendix S1: Supporting Information [file JSP2-4-e1162-s001.docx]

# A Comprehensive Toolbox for Large Animal Studies of Intervertebral Disc Degeneration and Regeneration

Supplementary File - Table of Contents

[A Comprehensive Toolbox for Large Animal Studies of Intervertebral Disc Degeneration and Regeneration 1](#_Toc72486298)

[1. Studies employed to determine the variety of experimental methodologies reported for outcome evaluation. 2](#_Toc72486299)

[1) Flowchart of the comprehensive tool box 4](#_Toc72486300)

[1) Biomechanics of the IVD 6](#_Toc72486301)

[2) Specimen preparation and storage for the purpose of biomechanical testing 6](#_Toc72486302)

[3) Detailed histopathology section 7](#_Toc72486303)

[Fixation 7](#_Toc72486304)

[Decalcification 7](#_Toc72486305)

[Embedding and sectioning 8](#_Toc72486306)

[Mounting and staining 9](#_Toc72486307)

[Immunostaining considerations: 10](#_Toc72486308)

[4) Protocols for standard (immuno)stainings 13](#_Toc72486309)

[Hematoxylin/Eosin 13](#_Toc72486310)

[Picrosirius red/alcian blue 14](#_Toc72486311)

[Safranin O/fast green 15](#_Toc72486312)

[Collagen type I and II immunohistochemistry 16](#_Toc72486313)

[5) Alternative histological processing protocol for ovine IVDs 19](#_Toc72486314)

[6) Histological scoring schemes available on large animal models 20](#_Toc72486315)

[References 23](#_Toc72486316)

## Studies employed to determine the variety of experimental methodologies reported for outcome evaluation.

A sample of the most recent peer reviewed manuscripts employing four common large animal models (i.e. canine, caprine, ovine, porcine n=10 per species) to study intervertebral disc degeneration or therapeutic strategies in the past two decades was selected for analysis. The studies included are summarized below (Supplementary Table 1). The number of six main outcome measures concomitantly used (macroscopic, histologic, radiologic, biochemical, biomechanical, pain) and the detailed use of each type of outcome were registered.

| **Animal** | | **PMID** | **Year** | **Journal** | **doi** |
| --- | --- | --- | --- | --- | --- |
| canine | 29899873 | | 2018 | Oncotarget | 10.18632/oncotarget.25476 |
|  | 30429487 | | 2018 | Sci Rep | 10.1038/s41598-018-35011-4 |
|  | 20839317 | | 2010 | J Orthop Res. | 10.1002/jor.21147 |
|  | 22849557 | | 2013 | Tissue Eng Part A | 10.1089/ten.TEA.2012.0255 |
|  | 19005697 | | 2008 | Eur Spine J | 10.1007/s00586-008-0750-6 |
|  | 18203202 | | 2008 | J Orthop Res. | 10.1002/jor.20584 |
|  | 19934809 | | 2009 | Spine | 10.1097/BRS.0b013e3181a54157 |
|  | 26013758 | | 2015 | Arthritis Res Ther | 10.1186/s13075-015-0625-2 |
|  | 26290179 | | 2015 | Arthritis Res Ther | 10.1186/s13075-015-0727-x |
|  | 30110616 | | 2018 | J Control Release | 10.1016/j.jconrel.2018.08.019 |
| ovine | 19521550 | | 2009 | Int J Biol Sci | 10.7150/ijbs.5.388 |
|  | 10973397 | | 2000 | Spine | 10.1097/00007632-200009010-00004 |
|  | 20521963 | | 2010 | Neurosurg Focus | 10.3171/2010.3.FOCUS1050 |
|  | 22404141 | | 2012 | J Neurosurg Spine | 10.3171/2012.1.SPINE11852 |
|  | 31283052 | | 2019 | J Orthop Res. | 10.1002/jor.24402 |
|  | 29055739 | | 2017 | Spine J | 10.1016/j.spinee.2017.10.008 |
|  | 26913464 | | 2016 | Spine | 10.1097/BRS.0000000000001528 |
|  | 26799116 | | 2016 | J Neurosurg Spine | 10.3171/2015.8.SPINE141097 |
|  | 22865642 | | 2014 | J Tissue Eng Regen Med | 10.1002/term.1582 |
|  | 19521550 | | 2009 | Int J Biol Sci | 10.7150/ijbs.5.388 |
| porcine | 21372649 | | 2011 | Spine | 10.1097/BRS.0b013e3181dce34c |
|  | 30478330 | | 2018 | Sci Rep | 10.1038/s41598-018-34582-6 |
|  | 21910592 | | 2011 | Tissue Eng Part A | 10.1089/ten.tea.2011.0229 |
|  | 17323162 | | 2007 | J Mater Sci Mater Med | 10.1007/s10856-006-0693-6 |
|  | 24801573 | | 2014 | Eur Spine J | 10.1007/s00586-014-3314-y |
|  | 19112334 | | 2009 | Spine | 10.1097/BRS.0b013e31818f8c20 |
|  | 20683387 | | 2011 | Spine | 10.1097/BRS.0b013e3181e08f01 |
|  | 20039083 | | 2010 | Eur Spine J | 10.1007/s00586-009-1255-7 |
|  | 31695783 | | 2019 | Theranostics | 10.7150/thno.34898 |
|  | 30509032 | | 2018 | Folia Neuropathol | 10.5114/fn.2018.76616 |
| caprine | 20890267 | | 2011 | Spine | 10.1097/BRS.0b013e3181d10401 |
|  | 17762288 | | 2007 | Spine | 10.1097/BRS.0b013e31811ebac5 |
|  | 32546053 | | 2020 | Tissue Eng Part A | 10.1089/ten.TEA.2020.0103 |
|  | 28735027 | | 2017 | Acta Biomater | 10.1016/j.actbio.2017.07.025 |
|  | 27568573 | | 2017 | Osteoarthritis Cartilage | 10.1016/j.joca.2016.08.006 |
|  | 26543683 | | 2015 | Biores Open Access | 10.1089/biores.2015.0025 |
|  | 25682272 | | 2015 | Eur Spine J | 10.1007/s00586-015-3803-7 |
|  | 23255234 | | 2012 | J Orthop Res. | 10.1002/jor.22296 |
|  | 21245789 | | 2011 | Spine | 10.1097/BRS.0b013e3181f60b39 |
|  | 20401620 | | 2010 | Eur Spine J | 10.1007/s00586-010-1384-z |

## Flowchart of the comprehensive tool box

**Flowchart** for processing the complementary read out parameters at the clinical, macroscopic and microscopic level. The recommended basic set of parameters is indicated in white boxes, while the optional ones are given in light blue boxes. Note that this basic set can be supplemented according to the research question.

1. Clinical grading applies to clinical studies employing veterinary patients or experimental animal studies evaluating disc-related pain. For the latter, the study design should address a single-disc level to allow for evaluation of disc-related pain.
2. We recommend image (T2-weighted or radiography) before induction of degeneration (baseline), at start of treatment (commonly 4-6 weeks after induction), and at the moment of euthanasia for macroscopic grading. Preferably, also a mid-term time point is recommended for longer studies, e.g. three months after initiation of treatment if the observation period lasts for six months. Basic imaging modalities can be combined with quantitative MR imaging (under the same anesthesia procedure)
3. *Postmortem* MR imaging (T2-weighted images or radiography and advanced imaging, e.g. CT scan) is possible. However, in the event that advanced quantitative MR imaging is used, it is recommended to do this prior to anesthesia as there are sequences that are influenced by changes in the core body temperature.
4. Here we also refer to advanced imaging methods described in our future perspectives
5. We generally recommend non-destructive biomechanical testing with fresh specimens. If storage is unavoidable, a description of optimal storage conditions can be found in the biomechanics section. Note that macroscopic and microscopic read outs might be affected as well by the storage method. Biomechanical testing may be performed with spinal specimens, FSUs, VCUs, CEP, NP or AF.
6. Note that macroscopy scoring should be done on both halves of the sagittally transected FSU.

## Biomechanics of the IVD

Six movements, *i.e.* degrees-of-freedom (DOF), can be described for the IVD in a 3-dimensional Cartesian coordinate system, with X dorso-ventral, Y lateral and Z cranial-caudal axes, according to ISO 2631 and Stokes *et al.*^1^. Left and right lateral bending is a pure rotation about the X-axis. Flexion (ventral bending) and extension (dorsal bending) is a pure rotation about the Y-axis. Axial rotation is a left or right rotation about the Z-axis. Compression or tension is a pure displacement in the -/+ Z-direction. Shear is defined as lateral, *i.e.* right and left displacement along the Y-axis or ventral and dorsal along the X-axis. The load-displacement curve (LDC) is a graphical illustration of the continuously changing load (force or moment) with changing deformation (displacement or rotation). The enclosed area between the loading and unloading curves, *i.e.,* hysteresis, represents the amount of lost energy (energy dissipation, *e.g.,* due to temperature increase) as a result of solid viscoelasticity and poroelasticity*, i.e.,* interstitial fluid flow. After approximately 3-10 cycles the hysteresis curve reaches steady-state^2,3^. The neutral zone (NZ) is the movement range of an LDC in which the specimen moves virtually across its neutral position/orientation with hardly any load. If a load above that within the NZ is applied, the deformation is described by the elastic zone (EZ), and it continues until the maximal (physiological) deformation is reached. The NZ and the EZ together are the range of motion (ROM), and when added bi-directionally is referred to as the combined ROM. The mechanical resistance to deformation can be described by the stiffness of the NZ and EZ, where both are measured by dividing the load by the deformation in a pre-described region of the LDC^3^.

## Specimen preparation and storage for the purpose of biomechanical testing

Whenever possible, fresh specimens are preferred for biomechanical testing. If specimens need to be stored, a 0.15 M PBS-soaked gauze can be placed around the unit and subsequently covered by at least two plastic bags/cling foils^4^ to prevent dehydration. Generally, mainly cartilaginous specimens (*i.e.* discs not containing NP notochordal cells) may be stored at 4 °C for at most overnight without a negative impact^5^. For a longer duration, specimens may be frozen, at -20 °C or lower for best sample preservation based on the available literature body (described in more detail in the Supplementary File). To date any additional benefits of storage at ultracold temperatures on IVD specimens have not been documented. Hydration is a pivotal aspect of disc mechanics and was shown to be unaltered due to freezing^6^. Furthermore, freezing of human disc specimens has been reported to not alter biomechanical behavior such as creep, intradiscal pressure, lateral bending or flexion, compared to fresh specimens^7–10^, and similar findings were reported for sheep discs^11^. For discs rich in notochordal cells (*i.e*., pig and NCD dogs), freezing is potentially more problematic. Several studies have demonstrated that the compressive behavior of pig IVDs is significantly changed due to freezing at -20°C^12–14^, and it may also damage the CEP^13^. However, Hongo *et al*^15^ showed that after initial changes due to freezing, no changes were observable in subsequent freeze-thaw cycles. Furthermore, they concluded that the high water content in these discs might lead to increased ice crystal formation and subsequently nuclear expansion^15^. In mixed-breed NCD dog discs, stiffness decreased after freezing at -80°C. However, the preparation for storage (*e.g.,* plastic bags) was not described in this study, which might have led to decreased water content^16^. To the best of our knowledge, there are no studies on the effects of freezing on the biomechanical behavior of CD dog discs as well as those of goats. Thus, we suggest treating NCD-dog discs similar to pig discs (notochordal cell-rich NP) and CD-dog and goat discs like sheep and human discs. For the retrieval and storage of disc tissues (CEP, NP, and AF) we suggest storing the entire intact IVD and harvesting the tissue directly before testing, if the tissue cannot be tested fresh. For future studies, one might also consider to freeze specimens comparable to cryopreservation for total disc transplantation^17^.

## Detailed histopathology section

Here, we describe common fixation and processing procedures and scoring systems for microscale analyses, providing technical and analytical considerations and potential drawbacks in their use.

### Fixation

For most applications, IVD tissues are fixed to preserve the cells and ECM and prevent autolysis. To minimize fixation time, the sample should be trimmed to the required size before fixation (Figure 1B; main manuscript). For example, an intact large animal FSU should be fixed for at least 1 week, with the fixative volume exceeding that of the tissue by a factor of 10. Note that the CEP needs to be maintained to prevent swelling of the NP tissue during fixation. In the recommended supplementary flowchart, fresh discs are transected mid-sagittally for further processing to collect macroscopic and microscopic outcomes. One limitation of this flow in processing is that healthy NP tissue will swell during fixation and as such it will not remain in the same plane with the other tissues of the processed specimen (*i.e*, AF, CEP, BEP). As a result of NP swelling, the tissue architecture is prone to distortion, and ECM components may leach out during the follow up tissue processing^18^. Instead, *en bloc* fixation can prevent these artefacts allowing for histopathological analysis of the IVD alone.

The recommended fixatives for IVD research purposes are 4% buffered formaldehyde or 10% neutral buffered formalin^19–21^. Cryo-compound fixation of samples immediately after removal of the CEPs can allow for fast processing with post-fixing after sectioning^22–24^. In combination with immunohistochemistry, ethanol fixation can avoid some of the antigen retrieval steps.

### Decalcification

As described in Figure 1 of the main manuscript, excess bone flanking cranial and caudal aspects of the adjacent vertebral body can be trimmed, sparing more bone adjacent to the EP if interested in bony changes (*e.g.* bone sclerosis, osteophytosis, Schmorl’s nodes, etc.).

Ethylenediaminetetraacetic acid (0.5 M EDTA) as a chelating agent is the method of choice for gentle decalcification, and generally the best at preserving tissue morphology^20,25^ and compatible with immunohistochemistry. Dependent on the amount of bone present, the species and age of animal, an extended period of decalcification may be needed (weeks to months), especially in large animal models. Decalcification can be accelerated through regular changes of the EDTA solution and by using gentle agitation.

Acid decalcification solutions, such as 5-10% solutions of hydrochloric acid, nitric acid, and formic acid^21,26^ can be used alone or in combinations if fast decalcification is required. However, some of the faster acid decalcifiers can result in severe tissue artifacts that produce a poorer tissue morphology and can impact subsequent staining. Care must be taken to minimize tissue destruction by regular controlling and immediate removal of the solution after the decalcification process. Alternative decalcification protocols that have been described include the incubation in EDTA under microwave heating to speed up the process, or the solution described by Kristensen, consisting of a buffer solution of equal parts of 8 N formic acid and 1 N sodium formate (pH 2.2)^27,28^. Furthermore, EDTA/formic acid has been employed for large animals with higher bone density such as goat and sheep to decrease decalcification time^29^. Aeration or agitation of the solutions can also help expedite the process. One of the alternative, shorter, protocols for decalcification compatible with histological staining and epitope retrieval methods is provided below (Alternative histological processing protocol for ovine IVDs).

Complete decalcification is essential to achieve high quality sections and maintain the tissue morphology. To monitor the decalcification process, the specimens are examined regularly (daily to weekly depending on decalcification process employed and the size of the sample) by micro-CT (µCT) or radiography (Supplementary Figure 1), depending on the available equipment. If radiologic equipment is not available to evaluate the decalcification process decalcification can also be checked by careful needle puncture (bony tissue needs to be as gentle as IVD tissue). There are also other methods to assess if the bony tissue is still mineralized, such as bending or cutting (also part of the physical testing), or chemical testing by Calcium oxalate test^30,31^.


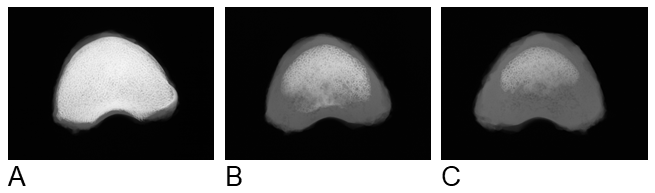


***Supplementary Figure 1. Radiographs of decalcification process:*** *Contact radiographs of ovine intervertebral disc during the decalcification process. (A) After 10 days; (B) after 6 weeks; (C) after 9 weeks of decalcification using 0,5M EDTA. Decalcification is complete when no (white) bone material is visible anymore.*

### Embedding and sectioning

Paraffin embedding is frequently used in research laboratories, as paraffin sections are suitable for a multitude of staining methods, including immunohistochemistry. Furthermore, paraffin microtomes are available in most laboratories, and the thin paraffin sections (5-10 µm) well preserve the cell and tissue morphology^19–21,28,32,33^.

IVD tissue is extremely challenging to process for paraffin embedding, since at each dehydration step, the morphology of the tissue tends to change. This is due to the fact that the NP retains its strong tendency to swell and expand, and therefore the tissues deform differentially. The difference in water content between the NP and AF results in comparatively more shrinkage and tissue deformation of the NP during alcohol dehydration, and greater shape changes when sectioned samples are floated on a water bath, as the NP has a greater tendency to absorb water. Furthermore, extra tissue trimming required to fit whole IVD tissue samples from large animals, including endplate and flanking vertebral bone, into standard paraffin molds may also contribute to NP expansion and additional structural deformities. Therefore, extra-large paraffin molds and blocks that can accommodate the larger average size of these specimens, and extra-large sample holders that are able to accommodate larger IVD tissue samples are recommended and are available from specialized producers. The aforementioned tissue distortions can result in technical challenges (*e.g*. reduced adherence of the sections to the slides), as well as produce section artefacts including tissue folds and clefts.

Resin embedding (*e.g.* using methyl-methacrylate) offers an attractive alternative to paraffin embedding^34–36^. Depending on the machine used and on the method of sectioning, the typical thickness of sections can vary from around 30 µm up to 900 µm (for very large samples with large implants). Thick sections can be created using a heavy-duty sledge (*e.g*. Polycut”), although, with a microtome thin sections (*e.g.* 5 µm thick) can be obtained. There are several advantages of resin embedding procedures: large specimens such as entire IVDs from large animals or humans can be processed in one piece; even metal implants or bone cement can be kept during the whole procedure; and no decalcification step is required, allowing evaluation of both mineralized and organic bone matrix. Moreover, the resulting sections preserve the original tissue structure and morphology in general very well and result in high quality histological images. One limitation of resin embedding and sectioning is the procedure requires special equipment and instruments not available in all laboratories, and personnel with specialized training. Moreover, to achieve optimal staining results in thick resin compared to thin paraffin sections, standard staining methods require modification. Finally, due to the embedding procedure that produces heat that inevitably alters the proteins and thus structures of most epitopes, immunohistochemical methods are difficult to apply; however, to the authors’ experience careful optimization immunohistochemistry protocols can be performed on thin (5-10 µm) sections.

### Mounting and staining

Tissue sections described above should be mounted on special positively-charged glass slides (*e.g.* SuperFrost Plus™). Many dyes used for section staining can be influenced by the fixative used. Hence, selecting the optimal fixative can improve staining. This is especially important for immunohistochemistry whereby the most appropriate fixative may be specified by the antibody protocol; thus selection of a fixative must be done prior to specimen processing. In routine and special histochemical stains, staining intensity and quality are impacted by many factors, including architectural and chemical features of the tissue (*e.g.* innate positive and negative charges that form bonds between the dyes, pore sizes within the tissue), chemical features of the stain (*e.g.* the particle size of the dye, concentration and pH of the dye solution), fixative used (in immunohistochemistry protocols, the most appropriate fixative is often specified by the antibody), exposure time to the tissues, and stain-to-tissue affinity. Hence, changing the pH of a solution or exposure time can dramatically impact staining intensity.

When evaluating histopathological sections of the IVD, it can be challenging to the unexperienced eye to discern between “true clefts,” *i.e*. lesions that arise from degeneration and separation or tearing of the matrix) versus artifactual clefts that arise from imperfect microtomy, *i.e*. “cutting artefacts.” The panel below highlights artifactual and true clefts identified in framed areas from Figure 7. Ancillary histochemical stains, such as Alcian blue-picrosirius red (AB-PSR), are helpful in conjunction with H&E to better characterize clefts and identify degenerative changes in the matrix that are often concurrent with true clefts. While cutting clefts tend to have sharp margins with abrupt transition to clear space without evidence of matrix degeneration (Grade 0, asterisk), true clefts are highlighted in the other panels. **Grade 1** panel has fine, poorly demarcated clefts spanned by degenerate (proteoglycan-rich), fibrillated matrix fibers interspersed with necrotic cells (**arrows**) concentrated within the ventral inner annulus. **Grade 2** panels contain fine fibrillations that arise within the inner to mid AF of the dorsal portion of the disc (**left**) that contains proteoglycan-rich degenerate matrix and rare necrotic cells (**arrow**); larger tears within the inner annulus of the ventral portion of the disc (**right**) also occur within regions of chondroid matrix metaplasia and are spanned by fibrillated matrix. **Grade 3** left panel contains irregularly interconnected rays of clefts spanned by fibrillated to coiled fibers in a background of proteoglycan-rich (**asterisks**) to proteoglycan poor matrix within the collapsed NP. Grade 3 right panel highlights true disruption of the cartilage endplate whereby extruded disc material demonstrates changes compatible with NP degeneration, characterized by proteoglycan-rich matrix interspersed with chondroid cells, *i.e*., chondroid metaplasia.


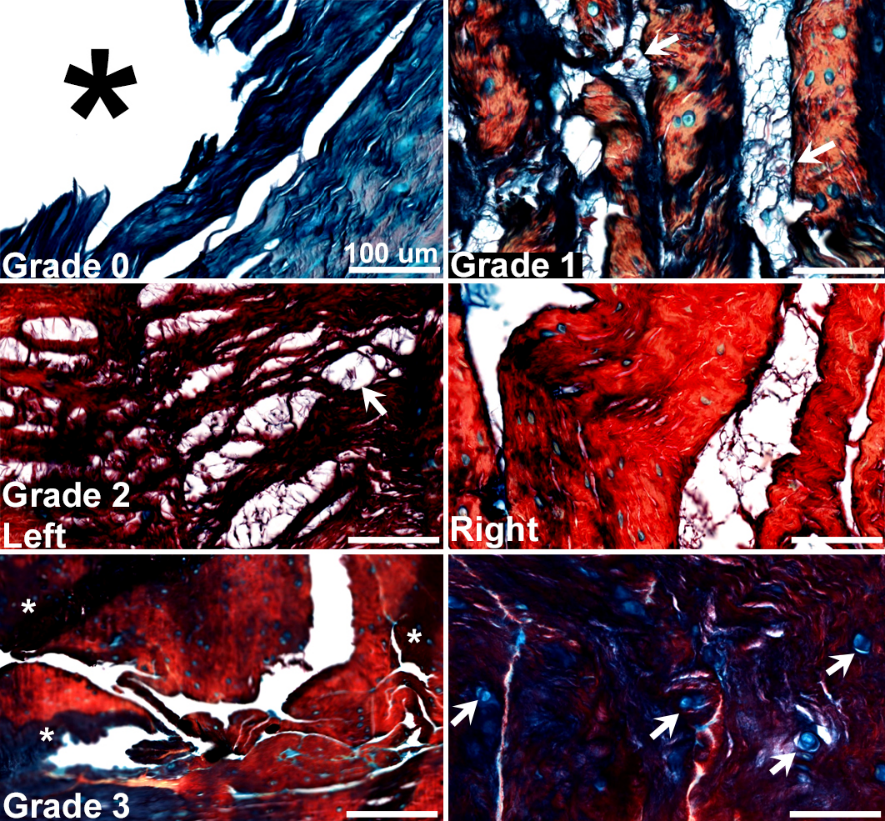


***Supplementary Figure 2 - composite depicting artifactual versus pathological clefts within the IVD highlighted in Figure 7A (bright-field microscopy, AB/PSR stain).***

*Artifacts of sectioning within the* ***Grade 0*** *NP are characterized by sharp, jagged, well- demarcated clefts (****asterisk****) occurring in a region with no evidence of degeneration.* ***Grade 1*** *IVD has fine, poorly demarcated clefts spanned by degenerate (proteoglycan-rich), fibrillated matrix fibers interspersed with necrotic cells (****arrows****) concentrated within the ventral inner annulus.* ***Grade 2*** *IVD contains fine fibrillations that arise within the inner to mid AF of the dorsal portion of the disc (****left****) that contains proteoglycan-rich degenerate matrix and rare necrotic cells (****arrow****); larger tears within the inner annulus of the ventral portion of the disc (****right****) also occur within regions of chondroid matrix metaplasia and are spanned by fibrillated matrix.* ***Grade 3*** *IVD contains irregularly interconnected rays of clefts spanned by fibrillated to coiled fibers in a background of proteoglycan-rich (****asterisks****) to proteoglycan poor matrix within the collapsed NP. Disc material extruded through the cartilage endplate contains proteoglycan-rich matrix interspersed with chondroid cells, compatible with NP degeneration.*

### Immunostaining considerations:

Antigen retrieval is frequently needed to expose the epitope for antibody binding. When citrate antigen retrieval is required, protocols may recommend temperatures in the range of 95-100°C. This can cause the NP tissue to detach from the slide, negating final tissue morphology. One way to mitigate the risk of detachment is to immerse the slides in a 37°C water bath and gradually increase the temperature, but not to above 70°C^37^. Note that using lower temperatures necessitates longer time for antigen retrieval.

The procedure of decalcification may affect epitope presentation, which can reduce the intensity of the immunostaining compared to non-decalcified tissue. In the author’s experience, immunostaining protocols that work for non-decalcified tissue sections often do not apply for decalcified sections. Recommended (immuno)stainings optimized for large animal IVDs are depicted in Supplementary table 1. Collagen type I and II are commonly used to evaluate ECM components of the IVD. Protocols with specific (immuno)staining examples are provided below. Note that 0.5 M EDTA-decalcified paraffin sections are the gold standard for these general (immuno)stainings, but that these stains can also be applied on sections that are decalcified in another way, *e.g.* the Kristensen method, are also applicable^38–40^ or EDTA/formic acid^29^. Appropriate negative (isotype control at the same concentration used as the primary target antibody) and positive controls (either within the tissue sectioned itself OR other tissue expressing the target protein) should be included for the purpose of demonstrating specificity of the antibody being employed.

| Staining | *Human* | *Dog* | *Pig* | *Goat* | *Sheep* |
| --- | --- | --- | --- | --- | --- |
| Hematoxylin and eosin | 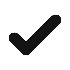^41^ | 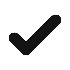^42^ | 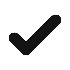^43^ | 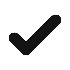^44–46^ | 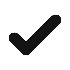^47–49^ |
| Picrosirius red/alcian blue | 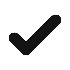^41^ | 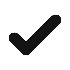^42^ | NA | 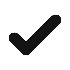^44–46^ | NA |
| Safranin O/fast green | 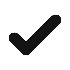^41,50^ | 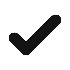^50^ | 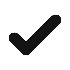^50,51^ | 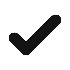^46^ | 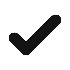^47^ |
| Collagen type I IHC | 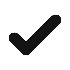^50,52^ | 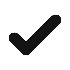^50,52^ | 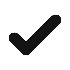^50^ | 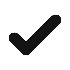^45^ | 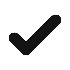^52^ |
| Collagen type II IHC | 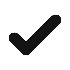^50,52^ | 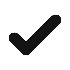^50,52^ | 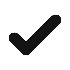^50^ | 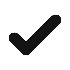^44,45^ | 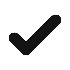^47^ |

**Supplementary table 2.** Recommended (immuno)stainings that have been set up for multiple large animal IVD tissues. Available protocols are described in the references and Supplementary File 2 (with specific staining examples for different species). NA: not available in literature (to the authors knowledge).

## Protocols for standard (immuno)stainings

### Hematoxylin/Eosin

Chemicals

- Hematoxylin solution
  - Mayers hematoxylin (Merck; 109249)
- Eosin solution
  - Eosin Yellowish (Merck; 115935): 0.2 gram
  - Ethanol 50%: 100 mL
  - Acetic acid (Boom; 51830): 1 drop)
- Depex (Merck; 100579)

Procedures

- Deparaffinize slides
  - 2 times Xylene (5 min)
  - 2 times 100% EtOH (3 min)
  - 2 times 96% and 70% EtOH (1 min
- Wash sections in MQ or PBS (5 min)
- Stain with Hematoxylin solution*
- Wash slides in running tap water (10 min)
- Stain the slides with Eosin solution (20 sec)
- Dehydrate sections
  - 2 times 70% and 96% (1 min)
  - 2 times 100% ethanol (3 min)
  - 2 times xylene (5 min)
- Mount with permanent mounting medium (Depex)

Note (*): Hematoxylin's staining intensity is dependent on the type of hematoxylin and staining duration. The protocols described here are based on Mayer's hematoxylin and Weigert's hematoxylin. Too intense Mayer's hematoxylin staining is frequently 'washed out’ with acidic solutions. However, intensity can also be decreased by decreasing the staining time. In combination with other stainings than eosin, Weigert's hematoxylin is frequently used, which needs a longer incubation time to stain.

### Picrosirius red/alcian blue

Chemicals

- 0.01 M HCl
- 0.5 mL 37% HCl (Merck; 100317)
- Weigert’s Hematoxylin
  - Mix an equal amount of Weigert’s solution 1 (Klinipath; 640495) and Weigert’s solution 2 (Klinipath; 640505)
- Alcian blue solution (pH 2.5)
  - 1 g Alcian blue (Sigma Aldrich; A5268)
  - 3 mL Glacial acetic acid (Boom; 76051830.1000)
  - 97 mL demi water
- Picrosirius red solution
  - 0.1 g Direct Red 80 (Sigma Aldrich; 365548)
  - 100 mL Saturated aqueous picric acid (Rie)
- Depex (Merck; 100579)

Procedures

- Deparaffinize slides
  - 2 times Xylene (5 min)
  - 2 times 100% EtOH (3 min)
  - 2 times 96% and 70% EtOH (1 min
- Wash sections in MQ or PBS (5 min)
- Stain the sections with Weigert’s hematoxylin (10 min)
- Rinse in running tap water (10 min)
- Stain in Alcian blue solution (30 min)
- Rinse in tap water (2 min)
- Stain in Picrosirius red solution (1 hour)
- Rinse in 0.01M HCl (2 min)
- Dehydrate sections
  - 2 times 70% and 96% (1 min)
  - 2 times 100% ethanol (3 min)
  - 2 times xylene (5 min)
- Mount with permanent mounting medium (Depex)

Note: In resin-embedded samples, Alcian blue/picrosirius red stain was found superior to other stains and stain combinations in terms of greater resolution of overall degeneration to cellular and matrix component including discernment of structural alterations across the entire IVD structure, and also had the greatest inter-rater agreement on the assigned degeneration scale^53^.

### Safranin O/fast green

Chemicals

- Weigert’s Hematoxylin
  - Mix an equal amount of Weigert’s solution 1 (Klinipath; 640495) and Weigert’s solution 2 (Klinipath; 640505)
- 1% Acetic Acid
  - 990 mL demi water
  - 10 mL Acetic acid (Boom; 76051830)
- 0.125% Safranin O
  - 100 mL demi water
  - 0.125 g Safranin O (Sigma; S8884)
  - This solution is typically made from a 1.25% (10x) Safranin O solution, 1.25g Safranin-O in 100 mL demi water, dilute this solution 1:10 in demi water
  - This solution can be used for multiple times, but not for multiple days
- Fast Green 0.4%
  - 0.4 g Fast Green (Sigma; F7252)
  - 100 mL demi water
  - This solution can be re-used until the quality of the green staining deteriorates
- Depex (Merck; 100579)

Procedures

- Deparaffinize slides
  - 2 times Xylene (5 min)
  - 2 times 100% EtOH (3 min)
  - 2 times 96% and 70% EtOH (1 min
- Wash sections in MQ or PBS (5 min)
- Stain with Weigert’s Hematoxylin (10 min)
- Rinse in running tap water (10 min)
- Stain with 0.4% aqueous Fast Green (4 min)
- Rinse in two changes of 1% Acetic Acid (3 and 2 min)
  - Slides should be cleared until color no longer runs
- Drain well
- Stain with 0.125% Safranin O (5 min)
- Dehydrate sections
  - 2 times 70% and 96% (1 min)
  - 2 times 100% ethanol (3 min)
  - 2 times xylene (5 min)
- Mount with permanent mounting medium (Depex)

Note: individuals who are red/green colour-blind may have difficulties evaluating these images. Toluidine blue/fast green is a useful alternative stain which works well in the IVD, as it has metachromatic properties with a shift to a purple colour when it interacts with GAGs. The purple-green colour combination also photographs better than Safranin O in black and white if colour photography is unavailable^18^.

### Collagen type I and II immunohistochemistry

Chemicals

- 0.3% H_2_0_2_
  - 1.72 mL 35% H_2_O_2_ (Boom; 76051810)
  - 198 mL PBS
- Hyaluronidase 10 mg/mL
  - 10 mg Hyaluronidase (Sigma; H3506)
  - 1 mL PBS
- Pronase 1 mg/mL
  - 1 mg Pronase (Roche; 11459643001)
  - 1 mL PBS
- Bright-DAB (Immunologic; VWRKBS04-110)
  - 1 mL of solution A ready to use buffered H_2_O_2_
  - 1 drop of solution B concentrated DAB solution
- Primary antibody collagen type I: mouse monoclonal antibody ab6308 (100 µg/mL)
  - Canine 1:1500, human: 1:1000, porcine: 1:1000, ovine: 1:1000
  - Negative control: normal mouse IgG_1_ (Dako; X0931)
- Primary antibody collagen type II: mouse monoclonal antibody DSHB, II-II6B3 (75 µg/mL)
  - Canine: 1:2500, human: 1:100, porcine: 1:100, ovine: 1:100
  - Negative control: normal mouse IgG_1_ (Dako; X0931)
- Secondary antibody
  - BrightVision Poly HRP anti-Mouse IgG (Immunologic VWRKDPVM110HRP)
- Mayers hematoxylin (Merck; 1.09249.0500)
- Depex (Merck; 100579)

Procedures

- Deparaffinize slides
  - 2 times Xylene (5 min)
  - 2 times 100% EtOH (3 min)
  - 2 times 96% and 70% EtOH (1 min
- Wash sections in PBS (5 min)
- Block with 0.3% H_2_0_2_ (10 min)
- Wash sections 2 times in PBS-T 0.1% (2 times 5 min)
- Antigen retrieval: Pronase 1 mg/mL (30 min @ 37°C)
- Wash sections 2 times in PBS-T 0.1% (2 times 5 min)
- Antigen retrieval: Hyaluronidase 10 mg/mL (30 min @ 37°C)
- Wash sections 2 times in PBS-T 0.1% (2 times 5 min)
- Block sections with PBS/BSA 5% (30 min)
- Incubate sections with Primary Antibody (overnight @ 4°C)
- Wash sections 2 times in PBS-T 0.1% (2 times 5 min)
- Incubate sections with secondary antibody conjugated with HRP (30 min @ RT)
- Wash sections 2 times with PBS (2 times 5 min)
- Incubate sections in Bright-DAB substrate kit (5 min)
- Rinse in demi water briefly (1 min)
- Counterstain with Mayers Hematoxylin (10-20 sec)
- Rinse in running tap water (10 min)
- Dehydrate sections
  - 2 times 70% and 96% (1 min)
  - 2 times 100% ethanol (3 min)
  - 2 times xylene (5 min)
- Mount with permanent mounting medium (Depex)


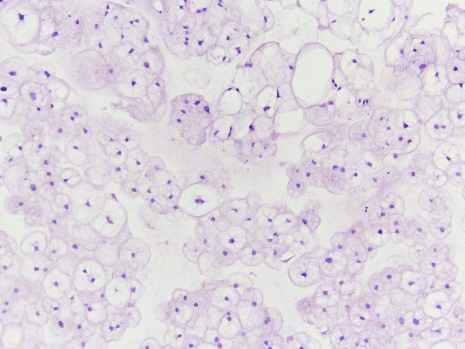

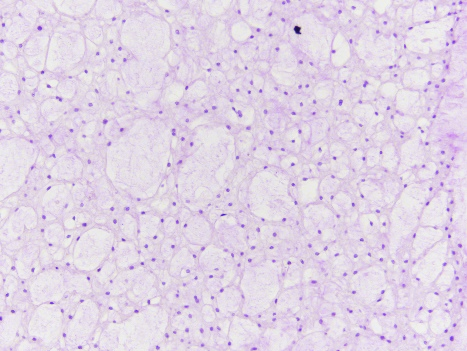

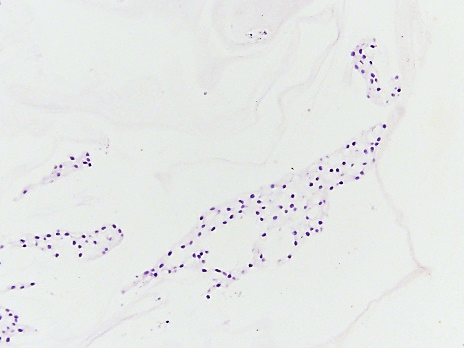


*Supplementary figure 3. Example of collagen type I immunostained canine, porcine and human fetal NP tissue with NCs (20 times magnification). The human NP mainly contains NPCs. Note that collagen type I IHC results should be negative in healthy NP tissue. Therefore, include negative control sections (e.g. AF tissue or TGF-β treated cell cultures).*


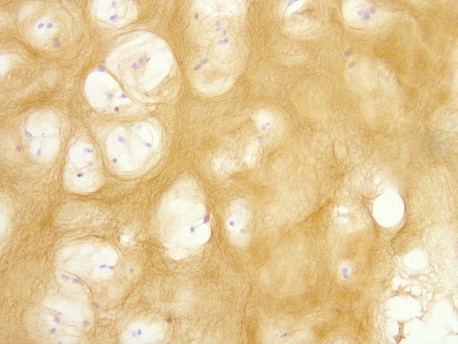

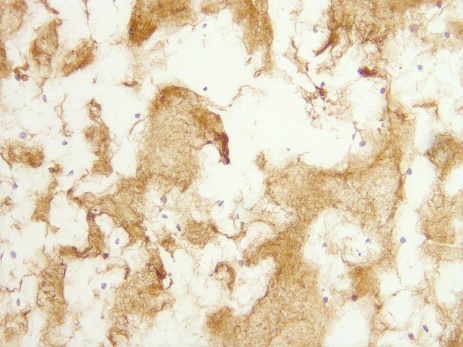

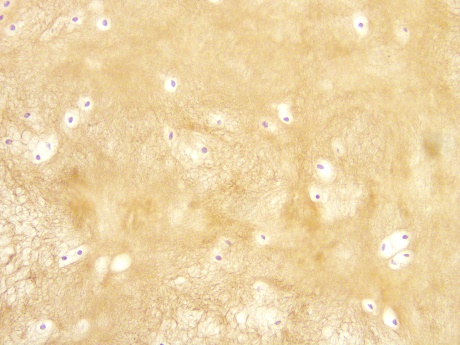


*Supplementary figure 4. Example of collagen type II immunostained canine, porcine and human fetal NP tissue with NCs (20 times magnification). The human NP mainly contains NPCs. Note that collagen type II IHC results should be positive in healthy NP tissue.*


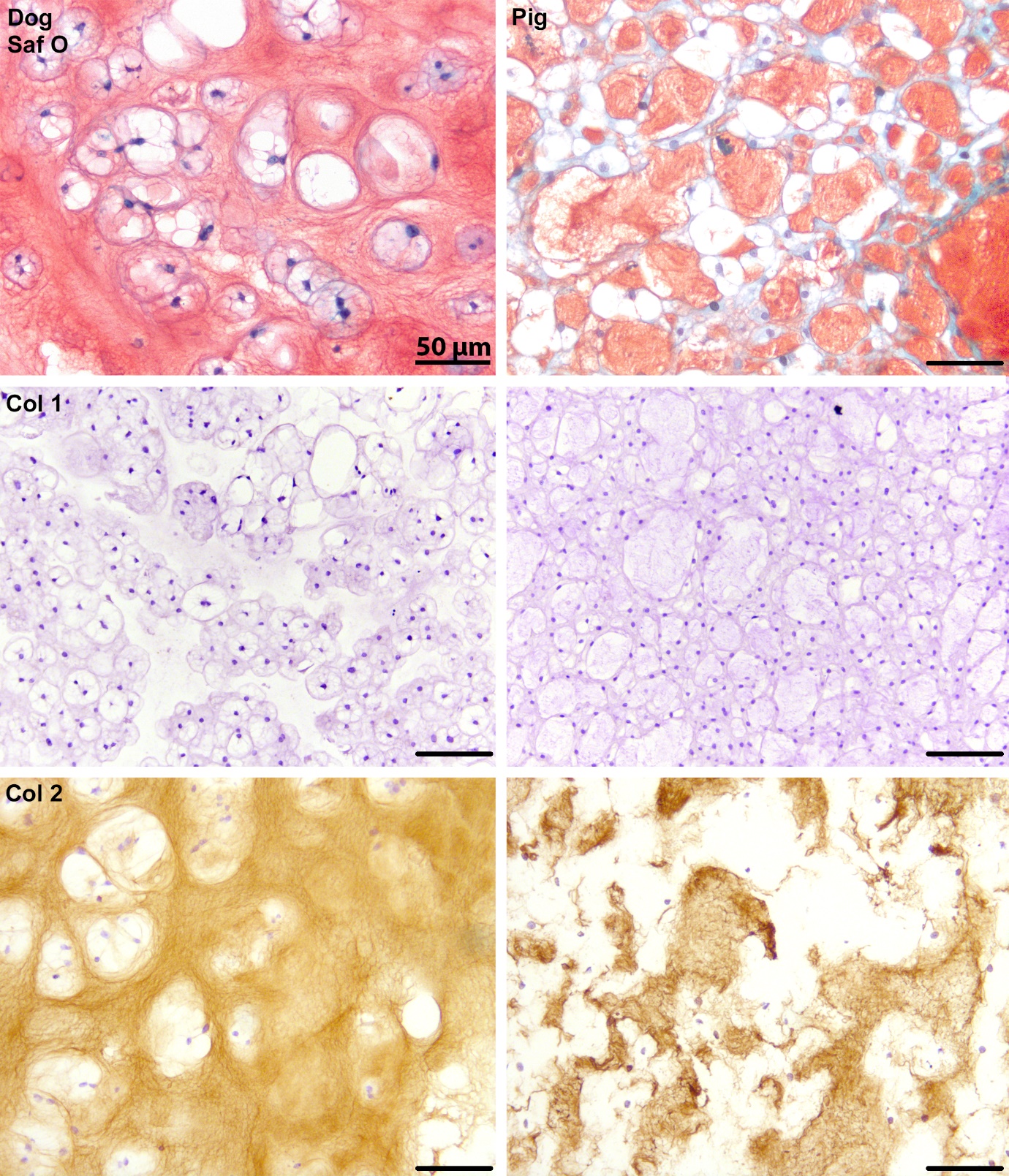
***Supplementary figure 5. Presence of notochordal cells in the nucleus pulposus and matrix staining for normal IVDs in the large animal models that have notochordal cell-rich discs.*** *Photomicrographs of dog (left panels) and pig (right panels). Upper panels show Safranin O/Fast Green (Saf O) stained sections that highlight the normal glycosaminoglycan (GAG)-rich extracellular matrix of the nucleus pulposus surrounding vacuolated notochordal cells. Middle panels show collagen type 1 (Col 1) immunohistochemical stained sections that demonstrate no collagen 1 staining within normal (i.e., non-degenerate) nucleus pulposus matrix. Lower panels show collagen type 2 (Col 2) immunohistochemical staining demonstrating a Collagen type 2-rich extracellular matrix within the normal nucleus pulposus.*

## Alternative histological processing protocol for ovine IVDs

Obtained from Raymond Purves Research Laboratories - Susan Smith

#### PROCESSING OF FSUs

Intervertebral disc (IVD) and vertebral body (VB) segments must be fixed, decalcified and dehydrated to 70%(v/v) ethanol before trimming or the disc will absorb water and expand beyond the edge of the bone. Trim the spinal segment to remove as much bone adjacent to the IVD with a bandsaw then a Dremel to remove the maximum amount of bone to minimise the depth of the block to facilitate faster penetration of fixative and decalcification solution. The trimmed IVD should be about 7-8 mm thick. Take care not to breach the IVD.

Fixation: Fix for 2 days in 10%(v/v) neutral buffered formalin in large volume containers (250mls) with agitation. Change fixative after 24 hours. Specimens may now be stored in 70%(v/v) ethanol.

Decalcification: Decalcify for 8 days in 10%(v/v) formic acid in 5%(v/v) formalin with agitation. Change the decalcifying solution every second day. Each IVD was decalcified in a 250ml container. Wash in water for 30 minutes then transfer to 70%(v/v) ethanol for 3 days. Change the ethanol each day. Trim the blocks to fit the histology cassettes.

#### HISTOLOGICAL PROCESSING OF IVD TISSUE

##### Dehydration

1. 75% (v/v) ethanol 5 hours
2. 85% (v/v) ethanol 5 hours
3. 95% (v/v) ethanol 5 hours
4. 100% (v/v) ethanol 4 hours
5. 100% (v/v) ethanol 4 hours
6. 100% (v/v) ethanol 4 hours

##### Clearing and infiltration with celloidin

Transfer specimen to enclosed containers.

1. Methyl benzoate 3 days
2. 1% (v/v) celloidin in methyl benzoate 3 days (or methyl benzoate only)
3. 4% (v/v) celloidin in methyl benzoate 7 days (or methyl benzoate only)

Rinse in 3 changes of chloroform, 10 minutes each to remove excess celloidin. (or 2 changes of chloroform 10 minutes each if methyl benzoate only was used)

Infiltration with Paraplast wax. Total 4 days.

1. Four times Paraplast wax 24 hours under vacuum; two changes.
2. Embed in Paraplast

Methyl benzoate has a very unpleasant odour and should be handled in a fume hood in enclosed containers.

Celloidin (a nitrocellulose compound derived from pyroxylin) is difficult to obtain now from chemical manufacturers and requires special importation and safety permits for its use as it is flammable and has been classified as a dangerous good, it is an explosion risk if dry. Trials without celloidin produce very nearly the same results as with celloidin. So it can be omitted. The time in methyl benzoate and wax are both important for the tissue to be fully infiltrated and therefore easier to cut with the microtome in thin 4 µm sections, producing a better IVD specimen with minimal artefacts. The IVD is a problematic tissue to process for histology and the steps indicated must be firmly adhered to.

Smaller IVDs from smaller animals will require less fixation and decalcification times but these procedures can be adapted proportionately from the above protocol.

| Histological scoring schemes available on large animal models | | | | | | | | | | | |  |  |
| --- | --- | --- | --- | --- | --- | --- | --- | --- | --- | --- | --- | --- | --- |
|  | **Dog** | **Pig** | | | **Sheep** | | | | **Goat** | **Human** | |  |  |
|  | *Bergknut 2013*^54^ | Barczewska 2018*^51^ | *Omlor 2009*^55^ | | *Shu*  *2017 ^&^* ^56^ | | *Kolf 2016*^49^ | | *Gullbrand 2017***^29^ | *Boos 2002*^57^ | *Rutges 2013*^41^ |  |  |
| **Disc degeneration** | naturally occurring | induced by percutaneous laser light deliveries | induced by partial nuclectomy | | induced by generating AF defects | | naturally occurring | | induced by chondroitinase ABC or subtotal nucleotomy | naturally occurring | naturally occurring |  |  |
|  | ***Annulus fibrosus morphology*** | | | | | | | | | | |  |  |
| Well-organized, half ring-shaped, collagen lamellae | 0 | x | ND | | 0 | | ND | | 0 | ND | 0 |  |  |
| Mild disorganized; some loss of half ring-shaped structure, most lamellar layer, still distinguishable (<25%) | 1 | x | ND | | 1 to 2 | | ND | | 1 | ND | ND |  |  |
| Moderately disorganized; partly ruptured AF, loss of half ring-shaped structure (25–75%) | 2 | x | ND | | 3 | | ND | | ND | ND | 1 |  |  |
| Completely ruptured AF; no or few distinguishable half ring-shaped collagen lamellae (>75%) | 3 | x | ND | | 4 to 5 | | ND | | 2 | ND | 2 |  |  |
|  | ***Chondrocyte metaplasia of annulus fibrosus*** | | | | | | | | | | |  |  |
| No chondrocyte morphology, just spindle-shaped fibroblasts | 0 | x | ND | | 0 | | ND | | ND | ND | ND |  |  |
| Mild chondrocyte proliferation (i.e. limited to inner most AF layers) | 1 | x | ND | | 1 | | ND | | ND | ND | ND |  |  |
| Moderate chondrocyte proliferation (i.e. chondroid cells in up to half of the AF) | 2 | x | ND | | 2 | | ND | | ND | ND | ND |  |  |
| Marked chondrocyte proliferation (i.e. chondroid cells up to outer layers of the AF) | 3 | x | ND | | 3 | | ND | | ND | ND | ND |  |  |
|  | ***Boundary between annulus fibrosus and nucleus pulposus*** | | | | | | | | | | |  |  |
| Clear boundary between AF and NP tissue | ND | ND | ND | | 0 | | ND | | 0 | ND | 0 |  |  |
| Boundary less clear; loss of annular-nuclear demarcation | ND | ND | ND | | 3 | | ND | | 1 | ND | 1 |  |  |
| No distinguishable boundary between AF and NP tissue | ND | ND | ND | | 5 | | ND | | 2 | ND | 2 |  |  |
|  | ***Tears and cleft formation in annulus fibrosus/nucleus pulposus*** | | | | | | | | | | |  |  |
| Absent | 0 | ND | 0 | | 0 | | 0 | | ND | 0 | ND |  |  |
| Rarely present | 1 | ND | 1 | | 1 to 4 | | 1 | | ND | 1 | ND |  |  |
| Present in intermediate amounts | 2 | ND | 1 | | 1 to 4 | | 2 | | ND | 2 | ND |  |  |
| Abundantly present | 3 | ND | 2 | | 1 to 4 | | 3 | | ND | 3 | ND |  |  |
| Scar/tissue defects | 4 | ND | 3 | | 5 | | 4 | | ND | 4 | ND |  |  |
|  | ***Nucleus pulposus cell proliferation*** | | | | | | | | | | |  |  |
| No proliferation | 0 | ND | 0 | | 0 | | 0 | | ND | 0 | 0 |  |  |
| Increased NPC density | 1 | ND | 1 | | 1 | | 1 | | ND | 1 | 1 |  |  |
| Connection of two NPCs | 2 | ND | 1 | | 1 | | 2 | | ND | 2 | 1 |  |  |
| Small size clones | 3 | ND | 2 | | 2 | | 3 | | ND | 3 | 1 |  |  |
| Moderate size clones | 4 | ND | 3 | | 2 | | 4 | | ND | 4 | 2 |  |  |
| Huge clones | 5 | ND | 4 | | 3 | | 5 | | ND | 5 | 2 |  |  |
| Scar/tissue defects | 6 | ND | 5 | | 5 | | 6 | | ND | 6 | ND |  |  |
|  | ***Presence of notochordal cells in nucleus pulposus*** | | | | | | | | | | |  |  |
| Abundantly present (>50%) | 0 | ND | x | | ND | | ND | | ND | ND | ND |  |  |
| Present (1–50%) | 1 | ND | x | | ND | | ND | | ND | ND | ND |  |  |
| Absent | 2 | ND | x | | ND | | ND | | ND | ND | ND |  |  |
|  | ***Matrix staining of the nucleus pulposus with alcian blue/picrosirius red*** | | | | | | | | | | |  |  |
| Blue stain dominates | 0 | x (SafO) | ND | | 0 or 1 | | ND | | 0 | ND | 0 |  |  |
| Mixture of blue and red staining | 1 | x (SafO) | ND | | 2 or 3 | | ND | | 1 | ND | 1 |  |  |
| Red stain dominates | 2 | x (SafO) | ND | | 3 | | ND | | 2 | ND | 2 |  |  |
|  | ***Endplate morphology*** | | | | | | | | | | |  |  |
| Regular thickness; homogeneous structure | 0 | ND | 0 | | ND | | 0 | | 0 | ND | 0 |  |  |
| Slightly irregular thickness | 1 | ND | 1 | | ND | | 1 | | 1 | ND | 1 |  |  |
| Moderately irregular thickness | 2 | ND | 2 | | ND | | 2 | | ND | ND | ND |  |  |
| Severely irregular thickness with interruption of the endplate | 3 | ND | 3 | | ND | | 3 | | 2 | ND | 2 |  |  |
|  | ***New bone formation - Osteophytes*** | | | | | | | | | | |  |  |
| Absent | 0 | ND | ND | | ND | | 0 | | ND | ND | ND |  |  |
| Minor new bone formation | 1 | ND | ND | | ND | | 1 | | ND | ND | ND |  |  |
| Moderate amounts of new bone formation | 2 | ND | ND | | ND | | 1 | | ND | ND | ND |  |  |
| Abundant new bone formation; tendency towards bridging/complete bridging | 3 | ND | ND | | ND | | 2 | | ND | ND | ND |  |  |
|  | ***Subchondral bone sclerosis*** | | | | | | | | | | |  |  |
| No sclerosis (<2 × the thickness of the dorsal vertebral cortex) | 0 | ND | 0 | | ND | | 0 | | ND | ND | ND |  |  |
| Mild sclerosis (2–4 × the thickness of the dorsal vertebral cortex) | 1 | ND | 1 | | ND | | 1 | | ND | ND | ND |  |  |
| Moderate sclerosis (>4 × the thickness of the dorsal vertebral cortex) | 2 | ND | 2 | | ND | | 1 | | ND | ND | ND |  |  |
| Severe subchondral bone irregularities | 3 | ND | 3 | | ND | | 2 | | ND | ND | ND |  |  |
|  |  |  | |  | |  | |  |  |  |  |  |  |

*: no grading scheme, only included which items are discussed &: scoring was done cumulatively for all tissues, scoring system split for the separate categories described here **: 0, 50 and 100 converted here in 0, 1 and 2 ; x: discussed in manuscript ; ND: not discussed/not included in reported grading scheme

## References

1. Stokes IAF. 1994. Three-Dimensional Terminology of Spinal Deformity A Report Presented to the Scoliosis Research Society by The Scoliosis Research Society Working Group on 3-D Terminology of Spinal Deformity. Spine (Phila. Pa. 1976). 19(2):236–248.

2. Robi K, Jakob N, Matevz K, Matjaz V. 2013. The Physiology of Sports Injuries and Repair Processes. In: Current Issues in Sports and Exercise Medicine. p 43–86.

3. Wilke HJ, Wenger K, Claes L. 1998. Testing criteria for spinal implants: Recommendations for the standardization of in vitro stability testing of spinal implants. Eur. Spine J. 7(2):148–154.

4. Bezci SE, O’Connell GD. 2018. Osmotic Pressure Alters Time-dependent Recovery Behavior of the Intervertebral Disc. Spine (Phila. Pa. 1976). 43(6):E334–E340.

5. Qi J, Hu Z, Song H, et al. 2016. Cartilage storage at 4°C with regular culture medium replacement benefits chondrocyte viability of osteochondral grafts in vitro. Cell Tissue Bank. 17(3):473–479.

6. Pflaster DS, Krag MH, Johnson CC, et al. 1997. Effect of Test Environment on Intervertebral Disc Hydration. Spine (Phila. Pa. 1976). 22(2):133–139.

7. Panjabi MM, Krag M, Summers D, Videman T. 1985. Biomechanical Time-Tolerance of Fresh Cadaveric Human Spine Specimens. J. Orthop. Res. 3:292–300.

8. Dhillon N, Bass EC, Lotz JC. 2001. Effect of frozen storage on the creep behavior of human intervertebral discs. Spine (Phila. Pa. 1976). 26(8):883–888.

9. Smeathers JE, Joanes DN. 1988. Dynamic compressive properties of human lumbar intervertebral joints: A comparison between fresh and thawed specimens. J. Biomech. 21(5):425–433.

10. NACHEMSON A. 1960. Lumbar intradiscal pressure: Experimental studies on post-mortem material. ACTA Orthop. Scand. 43:1–104.

11. Gleizes V, Viguier E, Feron JM, et al. 1998. Effects of freezing on the biomechanics of the intervertebral disc. Surg. Radiol. Anat. 20(6):403–407.

12. CALLAGHAN JP, MCGILL SM. 1995. Frozen storage increases the ultimate compressive load of porcine vertebrae. J. Orthop. Res. 13(5):809–812.

13. Bass EC, Duncan NA, Hariharan JS, et al. 1997. Frozen storage affects the compressive creep behavior of the porcine intervertebral disc. Spine (Phila. Pa. 1976). 22(24):2867–2876.

14. Azarnoosh M, Stoffel M, Quack V, et al. 2017. A comparative study of mechanical properties of fresh and frozen-thawed porcine intervertebral discs in a bioreactor environment. J. Mech. Behav. Biomed. Mater. 69:169–177.

15. Hongo M, Gay RE, Hsu J-T, et al. 2008. Effect of multiple freeze-thaw cycles on intervertebral dynamic motion characteristics in the porcine lumbar spine. J. Biomech. 41(4):916–920.

16. Flynn J, Rudert MJ, Olson E, et al. 1990. The effects of freezing or freeze-drying on the biomechanical properties of the canine intervertebral disc. Spine (Phila. Pa. 1976). 15(6):567–570.

17. Xiao J, Huang YC, Lam SKL, Luk KDK. 2015. Surgical technique for lumbar intervertebral disc transplantation in a goat model. Eur. Spine J. 24(9):1951–1958.

18. Melrose J, Smith SM, Smith MM, Little CB. 2008. The use of Histochoice^TM^® for histological examination of articular and growth plate cartilages, intervertebral disc and meniscus. Biotech. Histochem. 83(1):47–53.

19. Vadala G, Russo F, Pattappa G, et al. 2015. A Nucleotomy Model with Intact Annulus Fibrosus to Test Intervertebral Disc Regeneration Strategies. Tissue Eng Part C Methods 21(11):1117–1124.

20. Wangler S, Menzel U, Li Z, et al. 2019. CD146/MCAM distinguishes stem cell subpopulations with distinct migration and regenerative potential in degenerative intervertebral discs. Osteoarthr. Cartil. 27(7):1094–1105.

21. Shu CC, Smith MM, Smith SM, et al. 2017. A Histopathological Scheme for the Quantitative Scoring of Intervertebral Disc Degeneration and the Therapeutic Utility of Adult Mesenchymal Stem Cells for Intervertebral Disc Regeneration. Int. J. Mol. Sci. 18(1049):31.

22. Lang G, Liu Y, Geries J, et al. 2018. An intervertebral disc whole organ culture system to investigate proinflammatory and degenerative disc disease condition. J Tissue Eng Regen Med 12(4):E2051–E2061.

23. Pirvu T, Blanquer SB, Benneker LM, et al. 2015. A combined biomaterial and cellular approach for annulus fibrosus rupture repair. Biomaterials 42:11–19.

24. Li Z, Lang G, Chen X, et al. 2016. Polyurethane scaffold with in situ swelling capacity for nucleus pulposus replacement. Biomaterials 84:196–209.

25. Vadala G, Russo F, Pattappa G, et al. 2015. A Nucleotomy Model with Intact Annulus Fibrosus to Test Intervertebral Disc Regeneration Strategies. Tissue Eng. - Part C Methods 21(11):1117–1124.

26. Zhou Z, Zeiter S, Schmid T, et al. 2018. Effect of the CCL5-Releasing Fibrin Gel for Intervertebral Disc Regeneration. Cartilage 11(2):169–180.

27. Kristensen HK. 1948. An improved method of decalcification. Stain Technol. 23(3):151–154.

28. Rutges J, Creemers LB, Dhert W, et al. 2010. Variations in gene and protein expression in human nucleus pulposus in comparison with annulus fibrosus and cartilage cells: potential associations with aging and degeneration. Osteoarthr. Cartil. 18(3):416–423.

29. Gullbrand SE, Malhotra NR, Schaer TP, et al. 2017. A large animal model that recapitulates the spectrum of human intervertebral disc degeneration. Osteoarthr. Cartil. 25(1):146–156.

30. Clayden EC. 1952. A discussion on the preparation of bone sections by the paraffin wax method with special reference to the control of decalcification. J. Med. Lab. Technol. 10(103).

31. Arnim SS. 1935. A method for preparation of serial sections of teeth and surrounding structures of the rat. Anat. Rec. 62(3):321–330.

32. Zhu S, Lu P, Liu H, et al. 2015. Inhibition of Rac1 activity by controlled release of NSC23766 from chitosan microspheres effectively ameliorates osteoarthritis development in vivo. Ann. Rheum. Dis. 74(1):285–293.

33. Li Z, Lang G, Karfeld-Sulzer LS, et al. 2017. Heterodimeric BMP-2/7 for nucleus pulposus regeneration-In vitro and ex vivo studies. J Orthop Res 35(1):51–60.

34. Peroglio M, Douma LS, Caprez TS, et al. 2017. Intervertebral disc response to stem cell treatment is conditioned by disc state and cell carrier: An ex vivo study. J Orthop Transl 9:43–51.

35. Li Z, Lezuo P, Pattappa G, et al. 2016. Development of an ex vivo cavity model to study repair strategies in loaded intervertebral discs. Eur Spine J 25(9):2898–2908.

36. Long S.J.; Benneker, L.M.; Sakai, D.; Li, Z.; Pandit, A.; Grijpma, D.W.; Eglin, D.; Zeiter, S.; Schmid, T.; Eberli, U.; Nehrbass, D.; Di Pauli von Treuheim, T.; Alini, M.; Iatridis, J.C.; Grad, S. RG. F. 2020. Morphological and biomechanical effects of annulus fibrosus injury and repair in an ovine cervical model. JOR Spine 3(1):e1074.

37. Bach FC, Zhang Y, Miranda-Bedate A, et al. 2016. Increased caveolin-1 in intervertebral disc degeneration facilitates repair. Arthritis Res. Ther. 18(1).

38. Willems N, Mihov G, Grinwis GCM, et al. 2017. Safety of intradiscal injection and biocompatibility of polyester amide microspheres in a canine model predisposed to intervertebral disc degeneration. J. Biomed. Mater. Res. Part B Appl. Biomater. 105(4):707–714.

39. Willems N, Bach FC, Plomp SGM, et al. 2015. Intradiscal application of rhBMP-7 does not induce regeneration in a canine model of spontaneous intervertebral disc degeneration. Arthritis Res. Ther. 17(137):1–14.

40. Tellegen AR, Willems N, Beukers M, et al. 2017. Intradiscal application of a PCLA-PEG-PCLA hydrogel loaded with celecoxib for the treatment of back pain in canines: What’s in it for humans? J. Tissue Eng. Regen. Med. 12(3):642–652.

41. Rutges JPHJ, Duit RA, Kummer JA, et al. 2013. A validated new histological classification for intervertebral disc degeneration. Osteoarthr. Cartil. 21(12):2039–2047.

42. Bach FC, Tellegen AR, Beukers M, et al. 2018. Biologic canine and human intervertebral disc repair by notochordal cell-derived matrix: From bench towards bedside. Oncotarget 9(41):26507–26526.

43. Sheyn D, Ben-David S, Tawackoli W, et al. 2019. Human iPSCs can be differentiated into notochordal cells that reduce intervertebral disc degeneration in a porcine model. Theranostics 9(25):7506–7524.

44. Gullbrand SE, Schaer TP, Agarwal P, et al. 2017. Translation of an injectable triple-interpenetrating-network hydrogel for intervertebral disc regeneration in a goat model. ACTA Biomater. 60:201–209.

45. Gullbrand SE, Ashinsky BG, Bonnevie ED, et al. 2018. Long-term mechanical function and integration of an implanted tissue-engineered intervertebral disc. Sci. Transl. Med. 10(468).

46. Paul CPL, De Graaf M, Bisschop A, et al. 2017. Static axial overloading primes lumbar caprine intervertebral discs for posterior herniation. PLoS One 12(4).

47. Christian Woiciechowsky AA, Zenclussen ML, Casalis P, et al. 2014. Regeneration of nucleus pulposus tissue in an ovine intervertebral disc degeneration model by cell-free resorbable polymer scaffolds. J Tissue Eng Regen Med 18:811–820.

48. Melrose J, Smith SM, Little CB, et al. 2008. Recent advances in annular pathobiology provide insights into rim-lesion mediated intervertebral disc degeneration and potential new approaches to annular repair strategies. Eur. Spine J. 17(9):1131–1148.

49. Kolf AK, Hesper T, Schleich C, et al. 2016. T2∗ mapping of ovine intervertebral discs: Normative data for cervical and lumbar spine. J. Orthop. Res. 34(4):717–724.

50. Bach FC, de Vries SAH, Krouwels A, et al. 2015. The species-specific regenerative effects of notochordal cell-conditioned medium on chondrocyte-like cells derived from degenerated human intervertebral discs. Eur. Cells Mater. 30:132–147.

51. Barczewska M, Jezierska-Wozniak K, Habich A, et al. 2018. Evaluation of regenerative processes in the pig model of intervertebral disc degeneration after transplantation of bone marrow-derived mesenchymal stem cells. Folia Neuropathol. 56(2):124–132.

52. Bach FC, Laagland LT, Grant MP, et al. 2017. Link-N: The missing link towards intervertebral disc repair is species-specific. PLoS One 12(11):e0187831.

53. Walter BA, Torre OM, Laudier D, et al. 2015. Form and function of the intervertebral disc in health and disease: A morphological and stain comparison study. J. Anat. 227(6):707–716.

54. Bergknut N, Meij BP, Hagman R, et al. 2013. Intervertebral disc disease in dogs - part 1: a new histological grading scheme for classification of intervertebral disc degeneration in dogs. Vet. J. 195(2):156–163.

55. Omlor GW, Nerlich AG, Wilke HJ, et al. 2009. A new porcine in vivo animal model of disc degeneration: Response of anulus fibrosus cells, chondrocyte-like nucleus pulposus cells, and notochordal nucleus pulposus cells to partial nucleotomy. Spine (Phila. Pa. 1976). 34(25):2730–2739.

56. Shu CC, Smith MM, Smith SM, et al. 2017. A histopathological scheme for the quantitative scoring of intervertebral disc degeneration and the therapeutic utility of adult mesenchymal stem cells for intervertebral disc regeneration. Int. J. Mol. Sci. 18(1049):31.

57. Boos N, Weissbach S, Rohrbach H, et al. 2002. Classification of age-related changes in lumbar intervertebral discs: 2002 Volvo award in basic science. Spine (Phila. Pa. 1976). 27(23):2631–2644.
